# Synaptic dysfunction and extracellular matrix dysregulation in dopaminergic neurons from sporadic and E326K-*GBA1* Parkinson’s disease patients

**DOI:** 10.1038/s41531-024-00653-x

**Published:** 2024-02-19

**Authors:** Idan Rosh, Utkarsh Tripathi, Yara Hussein, Wote Amelo Rike, Jose Djamus, Boris Shklyar, Andreea Manole, Henry Houlden, Jurgen Winkler, Fred H. Gage, Shani Stern

**Affiliations:** 1 Sagol Department of Neurobiology, University of Haifa, Haifa, Israel; 2 Bioimaging Unit, Faculty of Natural Sciences, University of Haifa, Haifa, Israel; 3 Laboratory of Genetics, Gage, Salk Institute for Biological Studies, La Jolla, CA USA; 4 UCL Queen Square Institute of Neurology, University College London, London, England; 5 University Hospital Erlangen, Erlangen, Germany

**Keywords:** Parkinson's disease, Induced pluripotent stem cells, Synaptic transmission, Parkinson's disease, Reprogramming

## Abstract

Parkinson’s disease (PD) is a neurodegenerative disease with both genetic and sporadic origins. In this study, we investigated the electrophysiological properties, synaptic activity, and gene expression differences in dopaminergic (DA) neurons derived from induced pluripotent stem cells (iPSCs) of healthy controls, sporadic PD (sPD) patients, and PD patients with E326K-*GBA1* mutations. Our results demonstrate reduced sodium currents and synaptic activity in DA neurons derived from PD patients with E326K-*GBA1* mutations, suggesting a potential contribution to PD pathophysiology. We also observed distinct electrophysiological alterations in sPD DA neurons, which included a decrease in synaptic currents. RNA sequencing analysis revealed unique dysregulated pathways in sPD neurons and E326K-*GBA1* neurons, further supporting the notion that molecular mechanisms driving PD may differ between PD patients. In agreement with our previous reports, Extracellular matrix and Focal adhesion pathways were among the top dysregulated pathways in DA neurons from sPD patients and from patients with E326K-*GBA1* mutations. Overall, our study further confirms that impaired synaptic activity is a convergent functional phenotype in DA neurons derived from PD patients across multiple genetic mutations as well as sPD. At the transcriptome level, we find that the brain extracellular matrix is highly involved in PD pathology across multiple PD-associated mutations as well as sPD.

## Introduction

Parkinson’s disease (PD) is a neurodegenerative disease characterized by progressive extrapyramidal motor dysfunction^1^ and is considered to be an age-related disease^2–5^. The early stage and prodromal PD often goes undiagnosed in many patients until the later more severe appearance of motor dysfunction^6–8^. The development of PD motor deficits is associated with a progressive loss of dopaminergic neurons in the substantia nigra pars compacta and the subsequent depletion of dopamine levels in the striatum^9^. Albeit predominantly dependent on the presence of motor deficits such as bradykinesia, rigidity, and tremor for its diagnosis, PD is characterized by a range of non-motor preclinical features such as rapid eye movement sleep behavior disorder, anxiety disorders, anemia, depression, and constipation. Most interestingly, these non-motor symptoms, which are associated with abnormalities of the serotonergic, noradrenergic, and cholinergic systems, can, in some cases, manifest years before the disease onset and are linked to pathology in various parts of the nervous system^10–12^. With that said many aspects of PD’s etiology are still considered enigmatic, even two centuries after its initial description by Dr. James Parkinson^13^. Disease-modifying agents for PD are an unmet requirement, although it is still possible to alleviate symptoms with multiple types of medication, thus allowing PD patients to maintain their active life for several years longer^14,15^.

The majority of PD cases, about 85%, are considered a non-genetic disorder, i.e., with a ‘sporadic’ origin. Around 15% of PD cases are known to be inheritable and attributable to mutations in distinct sets of genes, sometimes in a specific genetic locus, and are considered to be familial PD^16–19^. Patients with the monogenic mutations are at an increased risk for PD and usually, the disease is developed at an early age. These genetic origins have different etiologies and progression of the disease and overall PD is a heterogenous disease but what is shared are the motor deficits. Some patients, however, have a slow progression of the disease and this varies in association with distinct carried risk factors^20,21^. In contrast, sPD is often characterized by a late-age onset, and sPD patients usually manifest motor symptoms after the age of 60–65. A minority of sPD patients present Parkinsonian symptoms before 40–50 years of age and are classified as cases of early onset. Apart from the age of onset, patients with sPD may have a different disease progression, clinical features, and response to medication^19,22^.

Several mechanisms have been suggested to be involved in the etiology of sPD, including abnormal handling of misfolded proteins by proteasomal enzymatic activities and autophagy, dysfunction in the mitochondrial electron transport chain, increased oxidative stress, and other pathogenic dysfunctions^16,17^. Pathologically, sPD, similar to many familial PD cases, is often characterized by the accumulation of α-synuclein in Lewy bodies and the degeneration of DA neurons in the substantia nigra pars compacta^23^. Recent studies of sPD cases have demonstrated alterations in synaptic activity and dysregulated extracellular matrix pathways in midbrain neurons derived from PD patients^24,25^.

The motor symptoms of sPD appear when putamenal DA is depleted around 80%, and around 60% of sunstantia nigra pars compacta DA neurons have already been lost, implying a possible involvement of a compensatory mechanism in the early stages of the disease^26^. However, most sPD patients lack a definitive genetic basis, making it challenging to create experimental models and find well-targeted medicines for disease management. Despite the lack of a clear genetic basis, numerous single nucleotide variants have been identified as contributing risk factors for sPD, suggesting that the condition might stem from a correlative composition of causative genetic factors^27–31^. Studies indicate that 5–15% of sPD patients carry β-glucocerebrosidase (*GBA1*) heterozygous gene mutations, making them the most prevalent genetic risk factor for sPD to be identified^32–35^. With the exception of an earlier age of onset, rapid progression of motor symptoms, and higher cognitive dysfunction, GBA-PD is not clinically distinct from sPD^32,36–39^. Furthermore, both conditions involve identical pathology, including nigrostriatal dopamine loss and the deposits of aggregated α-synuclein in the form of Lewy bodies^40–42^.

The *GBA1* gene encodes glucocerebrosidase (GCase), a lysosomal enzyme responsible for the cleavage of glucose from glucosylceramide (GlcCer) and glucosyl sphingosine (GlcSph)^43^. Most mutations in both alleles of *GBA1* result in Gaucher’s disease (GD), an autosomal recessively inherited lysosomal storage disease^20^. *GBA1* gene mutations have also been found to be significantly more common in PD patients than in non-affected individuals in over 50 population studies that have looked at the *GBA1* gene in PD patients^44^. Of the *GBA1* mutations, two-point mutations, N370S (p.N409S) and L444P (p.L483P) are the most common GD-associated ones^43,45^. These mutations vary across different populations, with N370S being the most common mutation among Ashkenazi Jews (AJ), accounting for about 70% of mutant alleles, and L444P the most common mutation among non-AJ European ancestors^46^. The E326K mutation in the *GBA1* gene is a specific point mutation and non-pathogenic polymorphism that has been implicated in an increased risk for PD and other synucleinopathies. Mutations in the *GBA1* gene, including the E326K variant, have been associated with reduced enzymatic activity and the subsequent accumulation of glycolipids in cells, leading to cellular dysfunction and neurodegeneration^32,46,47^.

The frequency of *GBA1* mutation carriers is high: 10–31% among the European AJ population compared to the European non-AJ population, where it is 2.9–12%^32^. The discovery of the association between *GBA1* mutations and increased PD risk has led to significant insights and advancements in understanding PD’s pathogenesis^48,49^. Several methods have been employed to develop a mouse model of *GBA1*-linked PD, such as knocking out *GBA1* or introducing point mutations and chemically induced models^50^. Although each approach provides distinct benefits, there is no single optimal mouse model for *GBA1*-PD. Unfortunately; there are no mouse models for sPD. Patients’ neurons derived from induced pluripotent stem cells (iPSCs) represent good prospects for bridging this gap in seeking mechanisms of uncovering therapeutic approaches for PD neurodegeneration. The ability to generate patient-specific iPSCs and human neurons has dramatically enhanced the molecular understanding of genetically complex diseases through better mirroring of their phenotypic manifestations by using live brain tissue models^18^.

With the help of iPSC techniques, it has been shown that mutations in the *GBA1* gene result in disrupted mitochondrial function^51^, increased endoplasmic reticulum (ER) stress^52^, impaired lysosomal morphology and function^51,53^, decreased levels of cathepsin D^54^, enhanced aggregation of α-synuclein^55^, and up-regulation of monoamine oxidases (MAO) in DA neurons^56^. Additionally, research using iPSC-derived DA neurons from PD patients with mutant *GBA1* demonstrated that these neurons had abnormalities in calcium homeostasis, an increase in α-synuclein and GlcCer levels, and a marked reduction in protein levels and GCase enzyme activity when compared to isogenic controls^55^. Similar results were obtained from a twin study that looked at iPSC-derived DA neurons and discovered that *GBA1* mutations are correlated with an elevated α-synuclein level and impaired GCase activity^57^. As reviewed by Tran et al.^18^, although sPD accounts for a significant proportion of all cases of PD, there are not many iPSC studies of sPD^24,25,58^.

Our current study concentrated on the neurophysiology and transcriptional alterations in DA neurons derived from PD patients with E326K-*GBA1* mutations and sPD patients. Our results revealed distinct and shared dysregulated pathways in both E326K-*GBA1*-associated and sPD cases, some of which are shared with our previous reports for other PD-related mutations^24,25,58^. In E326K-*GBA1*-associated PD, we found a significantly reduced influx of sodium currents and hypo excitability in DA neurons as well as dysregulation in ECM-receptor interaction, focal adhesion, and more pathways. In sPD DA neurons, we observed a dysregulation in pathways such as the PI3K-Akt signaling pathway, several synapse pathways, ECM–receptor interaction, focal adhesion, and more. We also measured spontaneous synaptic activity and observed a significantly reduced rate of spontaneous excitatory postsynaptic currents (EPSCs) and smaller amplitudes of EPSCs in DA neurons derived from E326K-*GBA1* PD patients compared to healthy individuals. In sPD, the synaptic activity was also decreased compared to healthy controls. Overall, this study strengthens our previous studies connecting synaptic impairments and dysregulation of ECM and focal adhesion-related genes to PD.

## Results

Our study included ten iPSC lines—three healthy controls (two males aged 71 and 43 years and one female aged 66 years), three PD patients with an E326K-*GBA1* mutation (all males aged 59, 63, and 65 years (the last one was homozygous for the mutation)), and four sporadic PD (sPD) patients. The sPD group included four males aged 46, 51, 55, and 65 years at disease onset. To investigate the intrinsic properties and synaptic activity of these cells, we differentiated the iPSCs into DA neurons (see the “Methods” section) and performed whole-cell patch clamp recordings (see the “Methods” section).

### E326K-*GBA1* mutant DA neurons exhibit reduced synaptic activity and a reduction in the sodium currents

**Fig. 1:**
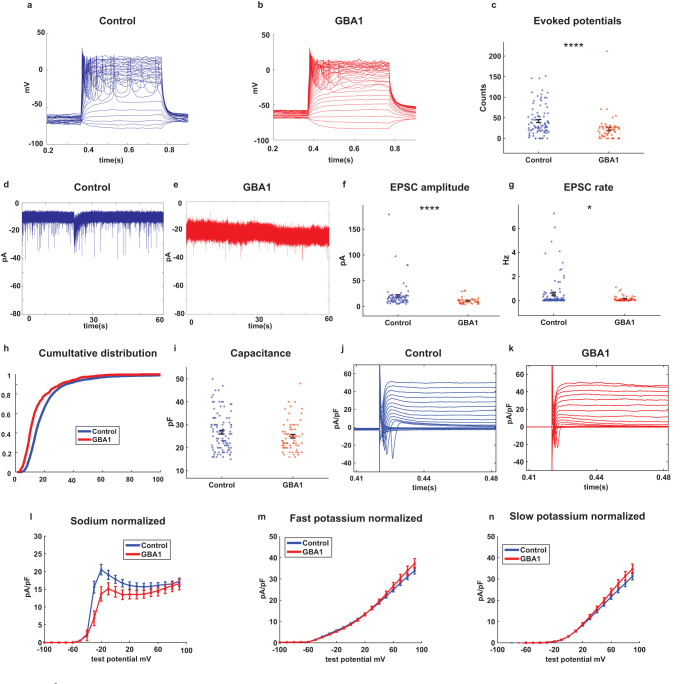
Reduced spontaneous synaptic activity and sodium currents in DA neurons derived from E326K-*GBA1* PD patients compared to healthy controls. 113 iPSC-derived DA neurons from three control individuals and 64 neurons from three PD patients with E326K-*GBA1* mutations were measured by whole-cell patch clamp. **a** and **b** Representative images of evoked action potentials recorded from DA neurons derived from a control and an E326K-*GBA1* PD patient, respectively. **c** The total number of evoked action potentials was significantly lower in E326K-*GBA1* mutant neurons compared to control neurons. **d** and **e** Example recordings of excitatory post-synaptic currents (EPSCs) in control and E326K-*GBA1* mutant neurons, respectively. **f** The mean amplitude of EPSCs was significantly reduced in E326K-*GBA1* mutant neurons compared to control neurons. **g** The EPSC rate was significantly lower in E326K-*GBA1* mutant neurons compared to control neurons. **h** The cumulative distribution of EPSC amplitudes of E326K-*GBA1* mutant and control neurons indicates lower EPSC amplitudes in E326K-*GBA1* mutant neurons. **i** No significant difference was observed in the capacitance of the E326K-*GBA1* and control neurons. **j** and **k** Examples of the recordings of sodium and potassium currents obtained in the voltage-clamp mode from control and E326K-*GBA1* mutant neurons, respectively. **l** Our findings indicate a significant decrease in the sodium currents between −20 to 0 mV in E326K-*GBA1* mutant neurons compared to control neurons. **m** No significant differences were observed in the fast potassium currents between 50 and 90 mV between E326K-*GBA1* mutant and control neurons. **n** No significant differences were observed in the slow potassium currents between 50 and 90 mV between E326K-*GBA1* mutant and control neurons. The results were acquired from 2 to 3 separate differentiation cycles for each of the cell lines. The sodium and potassium currents were analyzed using a one-way ANOVA, while all other comparisons were analyzed using the Mann–Whitney *U* test. Asterisks in this and the subsequent figures denote statistical significance as indicated by the following codes: *p < 0.05, **p < 0.01, ***p < 0.001, ****p < 0.0001. Error bars indicate the standard error of the mean (SEM).

Using a whole-cell patch-clamp, we recorded a total of 113 control and 64 E326K-*GBA1* mutant neurons from 3 control individuals and 3 E326K-*GBA1* PD patients. Figure 1a, b shows representative traces of evoked action potentials in control and E326K-*GBA1* mutant neurons, respectively. The total number of evoked potentials was significantly lower in E326K-*GBA1* mutant neurons compared to control neurons (Fig. 1c, *p* = 0.000049). Further, we compared the synaptic activity between control and E326K-*GBA1* mutant neurons measured by holding the neurons at a potential of −60 mV. Figure 1d, e show example recordings of EPSCs in control and E326K-*GBA1* mutant neurons, respectively. We observed a significant reduction in the mean amplitude of EPSCs in E326K-*GBA1* mutant neurons compared to control neurons (Fig. 1f, *p* = 0.000023). Additionally, the EPSC rate was significantly lower in E326K-*GBA1* mutant neurons compared to control neurons (Fig. 1g, *p* = 0.039). We also found the large EPSC rate in E326K-*GBA1* neurons to be significantly decreased compared to control neurons (Supplementary Fig. 1b, *p* = 0.000061). Figure 1h shows the cumulative distribution of EPSC amplitudes of E326K-*GBA1* mutant and control neurons. This distribution was left-shifted in the E326K-*GBA1* neurons compared to control neurons, indicating lower EPSC amplitudes. We observed no significant differences in the capacitance of E326K-*GBA1* compared to control neurons, indicating that the neurons’ total surface area was similar (Fig. 1i). The input conductance was not different between E326K-*GBA1* and control neurons (Supplementary Fig. 1d).

Figure 1j, k presents examples of the recordings of sodium and potassium currents obtained in the voltage-clamp mode from control and E326K-*GBA1* mutant neurons, respectively. Our findings indicate a significant reduction in the sodium currents between −20 and 0 mV in E326K-*GBA1* mutant neurons compared to control neurons (Fig. 1l, *p* = 0.0028). However, we observed no significant differences in the fast potassium and slow potassium currents between 50 and 90 mV in E326K-*GBA1* mutant neurons compared to control neurons (Fig. 1m, n, with a one-way ANOVA analysis). Overall, our results suggest that the E326K-*GBA1* mutation leads to a reduction in the sodium currents and synaptic activity in DA neurons.

### ECM receptor interaction, focal-adhesion, and pathways in cancer are amongst the strongest dysregulated pathways in E326K-*GBA1* mutant DA neurons

**Fig. 2:**
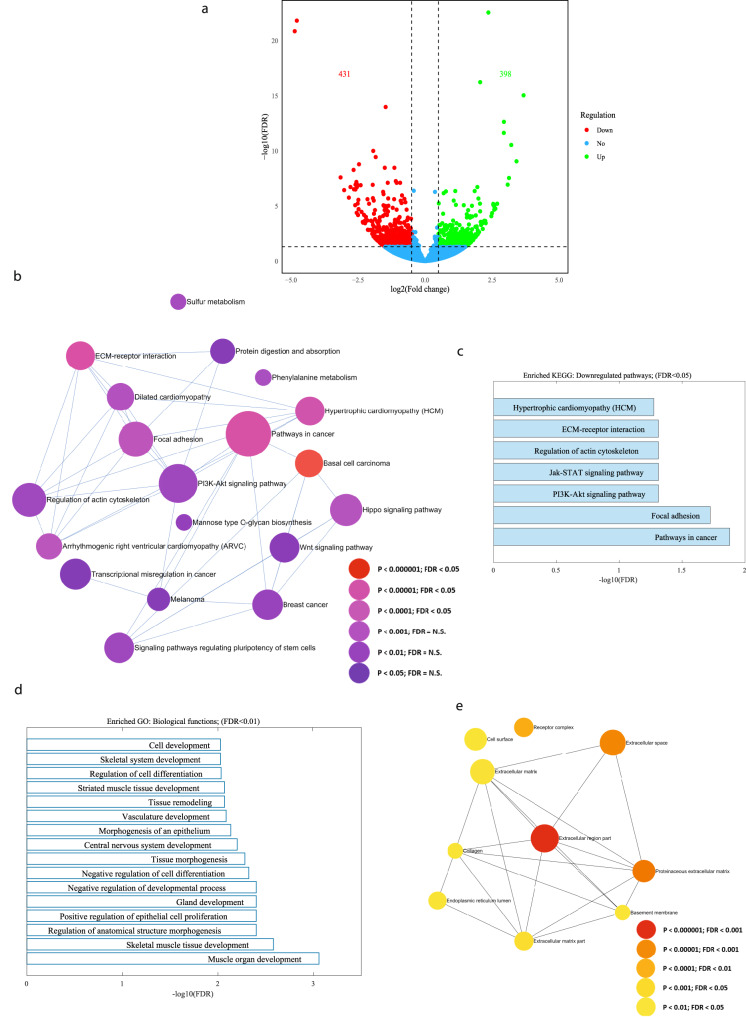
The top dysregulated pathways in E326K-*GBA1* mutant neurons relate to ECM, focal adhesion, and PI3K-Akt signaling. RNA was extracted from DA neurons derived from three E326K-*GBA1* PD patients and three healthy controls. **a** A volcano plot of DEGs between E326K-*GBA1* PD mutant DA neurons and healthy controls (|log2 fold change| > 0.5, FDR < 0.05). **b** Signaling network analysis with the top enriched KEGG pathways in E326K-*GBA1* PD mutant DA neurons compared to healthy controls (FDR < 0.05). The size of each node is proportional to the number of DEGs. **c** The top dysregulated (downregulated) KEGG pathways in the E326K-*GBA1* PD mutant DA neurons compared to healthy controls (FDR < 0.05). **d** The top dysregulated GO biological functions in the E326K-*GBA1* PD mutant DA neurons compared to controls (FDR < 0.01). **e** Signaling network analysis with the top enriched GO cellular components in the E326K-*GBA1* mutant DA neurons compared to healthy controls (FDR < 0.05).

Subsequently, RNA was extracted from DA neurons derived from three E326K-*GBA1* mutant PD patients and three control individuals, and gene expression differences were analyzed. Figure 2a illustrates a volcano plot of the differentially expressed genes (DEGs) between E326K-*GBA1* mutant neurons and controls. Specifically, 398 upregulated and 431 downregulated genes were identified in the E326K-*GBA1* DA neurons compared to healthy controls (|log2 fold change| > 0.5, FDR < 0.05). The signaling network analysis with the top enriched KEGG pathways of the E326K-*GBA1* mutant DA neurons compared to the controls is presented in Fig. 2b. The top dysregulated KEGG pathways are shown in Fig. 2c and include the ECM receptor interaction, focal adhesion, and Pathways in cancer. The ECM receptor interaction pathway plays a crucial role in the regulation of synaptic plasticity^59^, while focal adhesion proteins maintain the adhesion of brain cells to the ECM, which is crucial for neuronal migration and neurogenesis^60^ as well as cell survival^61,62^, and synaptic integrity^63^. Additionally, several developmental biological functions were dysregulated in the mutant group compared to the healthy control, as illustrated in Fig. 2d, such as Tissue morphogenesis and Tissue remodeling, which involve the organization and restricting of cells and ECM components^64^.

Furthermore, several extracellular matrix-related cellular components were dysregulated in the mutant group compared to healthy controls, as shown in Fig. 2e. Overall, similar to our previous reports^24,25^, we see that neurons derived from PD patients with different mutations have convergent dysregulated pathways, many of which are related to the ECM and genes that code for adhesion proteins of the cells to the ECM. We performed ICC to validate the expression level of collagen 4, one of the genes with downregulated expression and found that at the protein level, it was also downregulated (Supplementary Fig. 5a–j). Interestingly, collagen 4 formed larger aggregates in E326K-*GBA1* DA neuronal cultures (Supplementary Fig. 5i). The PI3K-Akt signaling pathway is also a convergent dysregulated pathway in PD. The entire list of dysregulated pathways can be found in Supplementary Tables 1-5.

### sPD DA neurons exhibit a decrease in synaptic activity

**Fig. 3:**
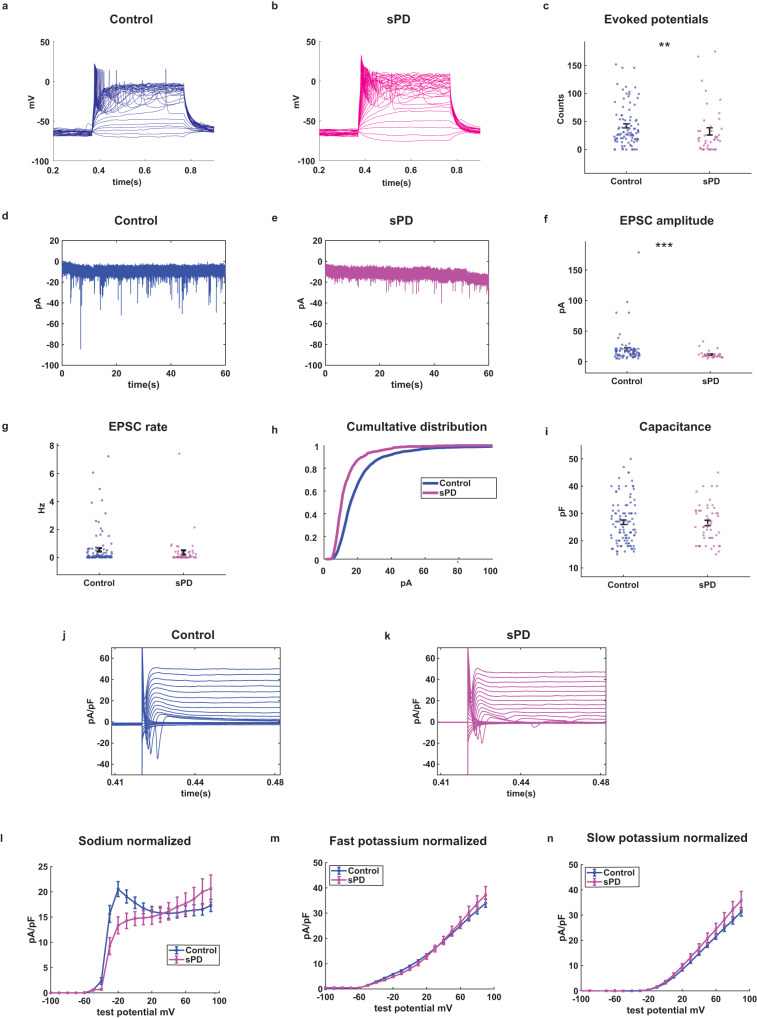
Synaptic dysfunction observed in DA neurons derived from patients with sPD compared to healthy controls. We recorded a total of 113 control and 57 sPD neurons derived from 3 control and 4 sPD patients. **a** and **b** Representative traces of evoked action potentials of control and sPD neurons, respectively. **c** sPD DA neurons were hypoexcitable compared to control neurons as measured by the total evoked action potentials. **d** and **e** Example recordings of excitatory post-synaptic currents (EPSCs) in control and sPD DA neurons, respectively. **f** Reduced amplitudes of EPSCs in sPD DA neurons as compared to control DA neurons. **g** The rate of EPSCs was not significantly different between sPD and control neurons. **h** The cumulative distribution of EPSC amplitudes in control and sPD neurons. **i** The capacitance was not significantly different between control and sPD neurons. **j** and **k** Examples recordings of the sodium and potassium currents obtained in voltage-clamp mode from a control and an sPD neuron. **l** Sodium currents were decreased in sPD DA neurons compared to controls. **m** Fast potassium currents and **n** slow potassium currents were not significantly different between sPD and control neurons. The results were acquired from 2 to 3 separate differentiation cycles for each of the cell lines. The sodium and potassium currents were analyzed using a one-way ANOVA, while all other comparisons were analyzed using the Mann–Whitney *U* test. Error bars indicate the standard error of the mean (SEM).

Using the whole-cell patch-clamp, we recorded a total of 113 control and 57 sPD neurons derived from 3 control and 4 sPD patients. Figure 3a, b provides representative traces of evoked action potentials in a control neuron and an sPD neuron. Our findings indicate that the excitability of the sPD neurons is decreased compared to control neurons. The average excitability is depicted in Fig. 3c (*p* = 0.008 for control vs. sPD). Figure 3d, e provides examples of EPSC recordings from a control and an sPD DA neuron measured in a voltage clamp of −60 mV. There was a significant decrease in the mean amplitude of EPSCs in sPD neurons compared to controls (Fig. 3f, *p* = 0.00097 for control vs. sPD neurons). We did not find any significant difference in the EPSC rate in sPD neurons compared to control neurons, (Fig. 2g, *p* = 0.11). However, a significant decrease in the EPSC rate was noted when comparing the large EPSCs (see the “Methods” section) (Supplementary Fig. 1a, *p* = 0.0042). This agrees with our previous report^24^ and indicates that the synaptic connectivity is reduced in sPD, but to a lower degree than most monogenic PD mutations. Figure 3h illustrates the cumulative distribution of EPSC amplitudes in sPD and control neurons. The cumulative distribution of EPSC amplitudes in sPD neurons was left-shifted compared to control neurons, suggesting lower EPSC amplitudes. To further validate the synaptic defects, we stained for Syanpsin1 and PSD95 and saw a dramatic decrease in synapsin1::PSD95 pairs in E326K-*GBA1* patient-derived neurons, and to a lesser extent in the sPD neurons (Supplementary Fig. 4). There was no significant difference between the capacitance (Fig. 3i) and input conductance (Supplementary Fig. 1c) between control and sPD neurons.

Figure 3j, k demonstrates examples of the recordings of sodium and potassium currents in voltage-clamp mode from control and sPD neurons. Our findings reveal that the sodium currents were significantly reduced in sPD neurons compared to control neurons (Fig. 3l, *p* = 0.003) for currents in the range of −20 to 0 mV. However, we observed no significant differences in the fast potassium and slow potassium currents recorded in potentials between 50 and 90 mV between sPD and control neurons (Fig. 3m, n).

### ECM and synaptic pathways are dysregulated in sPD DA neurons

**Fig. 4:**
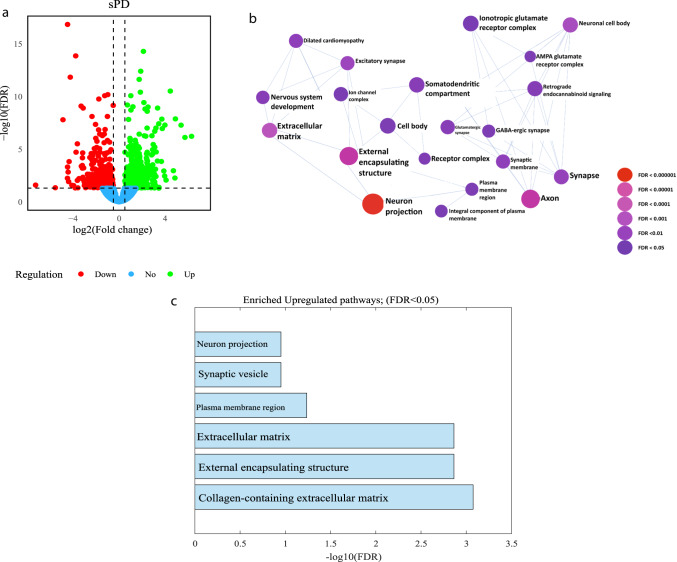
The top dysregulated pathways in sPD DA neurons relate to the Synapse and the ECM. RNA was extracted from DA neurons derived from three control individuals and two sPD patients. **a** A volcano plot of DEGs in sPD compared to controls (|log2 fold change| > 0.5, FDR < 0.05). **b** Signaling network analysis with the top enriched GO cellular components pathways in sPD DA neurons compared to healthy controls (FDR < 0.05), the size of each node is proportional to the number of DEGs. **c** The top dysregulated (upregulated) GO cellular components pathways in sPD DA neurons compared to healthy controls (FDR < 0.01).

Analysis of gene expression differences between DA neurons derived from sPD patients and controls was conducted (Fig. 4). Figure 4a shows a volcano plot of DEGs in sPD compared to healthy controls (|log2 fold change| > 0.5, FDR < 0.05) (412 genes were upregulated and 362 were downregulated in sPD compared to controls). The signaling network analysis with the top enriched gene ontology (GO) cellular components pathways in sPD DA neurons compared to healthy controls is presented in Fig. 4b. The top-upregulated GO cellular components pathways in sPD are shown in Fig. 4c and include several synapse-related pathways and ECM-related pathways. The entire list of dysregulated pathways can be found in Supplementary Tables 6–10. We performed ICC to validate the protein expression level of collagen 1, one of the genes with downregulated expression levels and found that at the protein level, it was also downregulated (Supplementary Fig. 5k–s).

## Discussion

Parkinson’s disease affects up to 3% of people over the age of 80 and also strikes people in their 30s and 40s. Its impact has far-reaching consequences for individuals, families, and society as a whole^65^. As a result, extensive research has been conducted, and several PD-causing mutations have been reported; however, the majority of PD cases have no known genetic cause and are classified as sPD cases^14^. While most sPD cases lack a definitive genetic cause, suggesting potential environmental contributions, there are challenges in recapitulating these putative environmental factors in iPSC models. This represents a limitation in modeling sPD, as important environmental exposures may be absent in the in vitro differentiation paradigm used in our current study. The phenotypes that we observed in iPSC-derived neurons suggest that there is a biological contribution that may arise not from a single gene but rather a concert of genes acting together in a way that is still not understood. Therefore, iPSC-derived neurons are helpful at least for assessing this contribution but cannot recapitulate environmental factors. Direct conversion models from fibroblasts to neurons^66^ may be able to shed light on environmental factors as well.

*GBA1* mutations have a significant impact on the pathogenesis of PD and have been reported to have a clear association with the disease^23,31^. We investigated the intrinsic and network properties of DA neurons derived using iPSC techniques from sPD patients and PD patients who are E326K-*GBA1* mutation carriers (heterozygous as well as homozygous for the E326K-*GBA1* mutation). We additionally analyzed the transcriptome and found some significant changes.

In this study, we had 3 controls, 4 sPD patients, and 3 PD patients with E326K-*GBA1* mutations. Except for one of the control lines, all the individuals were males. The individuals were adults in an age range of 46–71. The differences between male and female neurons have not been thoroughly investigated so it is important to keep in mind that one of the controls was a female. Regarding age, multiple studies suggest that reprogramming erases cellular aging and iPSC-derived neurons have no traces of the donor’s age^67,68^. Our findings suggest that the E326K mutation in the *GBA1* gene may lead to a reduction in the sodium currents and a decrease in the synaptic activity in DA neurons and these are also observed in sPD-derived neurons. Studies have shown that many DA neurons are also glutamatergic^69^ and exert rapid synaptic currents by their glutamatergic synapses and slower modulatory actions via their dopaminergic synapses. Therefore, EPSCs are expected to be present when recording synaptic currents in our DA neuronal cultures, and we have measured these to be different in these PD models.

We have previously reported alterations in synaptic activity as a shared phenotype of DA neurons derived from PD patients with other mutations^24,25,58^. It has been demonstrated that reprogramming adult cells into iPSCs removes aging and epigenetic modifications^67,68^. Thus, neurons in our cultures are young neurons. In light of this, the observed phenotype of decreased synaptic activity suggests an early mechanism that is present in the patients’ DA neurons long before the patients experience motor deficits. This finding is consistent with several studies using PD model mice, which suggested that the early phenotype is a decrease in synaptic activity that progressed to motor dysfunction in later stages^70,71^. These results also suggest that there is a yet unknown genetic mechanism that causes these phenotypes. Thus, monitoring synaptic activity may significantly impact the early diagnosis and prediction of PD onset. It is important to note that when performing ICC in the DA neuronal cultures, there was an increase in the percentage of cells that were positive for caspase3 in the E326K-*GBA1* lines. This means that already at this stage, there is an increased mortality in the cells and this should be considered when reporting the deficits in the synaptic currents. However, the overall density of the MAP2-positive cells did not significantly change, and therefore, the reduction in the synaptic currents is not likely to be due to neuronal loss. While most PD mutations cause a reduction in synaptic activity^24,25^, PINK1, together with PARK2 mutations, cause an increase in the synaptic activity of DA neurons^25^. Our current and previous^24^ findings suggest that sPD DA neurons’ synaptic activity is similar to most monogenic mutations but with a milder phenotype.

Transcriptome analysis and comparison between PD and control DA neurons indicate several intracellular and extracellular pathways that are dysregulated in PD. These include several ECM pathways that are commonly dysregulated in PD in this study and previous studies^24,25,72^. These findings suggest a focus on the ECM and its strong involvement in PD. Studies of the ECM composition and structure should be conducted as these are currently under-explored in PD^73,74^. The brain ECM has been shown to closely affect neurite outgrowth and connectivity during neural development^75^. In alignment with our observed dysregulation of ECM-related pathways and the connection to neurite outgrowth, Bogetofte et al. recently demonstrated neurite outgrowth impairments in proteomic analysis of N370S *GBA1* mutant DA neurons^76^. The synaptic deficits that are also a shared phenotype in PD suggest a temporal correlation between the synaptic impairment and the ECM deficits that needs further investigation in the context of PD. The diffuse perisynaptic matrix and the synaptic ECM of perineuronal nets (PNNs) together with the pre and post-synaptic neurons and glial cells, comprise the fourth compartment and recent addition of the tetrapartite synapse^77^. Additionally, pathways that relate to focal adhesion were dysregulated in the E326K-*GBA1* neurons as well as other PD lines in our previous reports. Many of the genes that were dysregulated in these pathways are various integrins. Integrins regulate synapse formation and maturation. They help with the regulation of postsynaptic strength by controlling the dynamics of neurotransmitter receptors and they additionally alter dendritic spines by actin remodeling^78^. It is therefore reasonable that changes in the ECM and focal adhesion proteins will affect synaptic connections and visa-versa, and our study emphasizes the need to study these in PD.

We have observed common dysregulated pathways between sPD and E326K-*GBA1* PD and also other mutations^24^. When we plotted the top dysregulated pathways, 50% of them were shared between sPD and E326K-*GBA1* mutant neurons. This is interesting in light of similar symptoms of the patients, yet there are differences in the genetics. Some of the common dysregulated intracellular signaling pathways are the PI3K-Akt signaling pathway, cancer-related pathways, and Hippo signaling, as examples (in the E326K-*GBA1* and our previous studies^24^). It is interesting to note that many types of cancers are associated with increased expression of matrisome genes and increased cell survival^79^. Our work in this study and previous studies^24,72,74^ shows that a decrease in matrisome genes is associated with PD and neurodegeneration. Dysregulation in cardiomyopathy pathways is also shared between different PD lines. Cardiomyopathy has been associated with PD^80^. It is important to keep in mind that this is a neuronal culture. However, cardiomyocytes are also excitable cells and also express some similar ion channels. Therefore, it is not surprising to see evidence of these changes also in neurons transcriptome. Further validation is needed by differentiating human cardiomyocytes. The intracellular pathways that are affected play essential roles in regulating various cellular processes, including cell growth, differentiation, proliferation, and survival. Also, dysregulation of these intracellular pathways has been found relevant in neurodevelopmental diseases and neurodegenerative diseases, including Parkinson’s disease^81–86^. For example, the Hippo signaling pathway is dysregulated in the E326K-*GBA1* mutant PD. This pathway is highly evolutionarily conserved and regulates cell proliferation, apoptosis, and stem cell self-renewal. Its activation plays a role in neurodegeneration by mediating oxidative stress-induced neuronal death^86^.”

It is also interesting to note that many of the more classical PD-related dysregulated molecular and cellular pathways do not appear in our list of dysregulated pathways. This is probably due to the fact that iPSC-derived neurons are young neurons and therefore allow us to see dysregulation that likely happens at the prodromal steps of the disease. The more classical pathways that were found include PI3K-Akt signaling pathway^87^, synaptic pathways^88^ – usually described as an early event, MAPK and Ras signaling pathways^89,90^, TGF-beta signaling pathway^91^, Inflammatory mediator regulation of TRP channels^92^, FoxO signaling pathway^93^, sulfur metabolism^94^, purine metabolism^95^, Phenylalanine metabolism^96^, and more.

We identified transcriptional changes related to neurodevelopmental and cell maturation pathways that were dysregulated in the patient-derived dopaminergic neurons compared to controls. As Parkinson’s disease has an adult-onset, altered expression of early developmental genes could reflect intrinsic ongoing maturation in the stem cell-derived neurons, which may not fully reach an adult-like state within the differentiation timeline. On the other hand, there have been few review studies talking about a neurodevelopmental aspect of PD^97,98^. The reduced sodium currents we observed in E326K-*GBA1* and sPD neurons compared to controls could indicate delayed maturation, as larger sodium currents have been associated with more mature neuronal states. Further time course analyses evaluating gene expression and electrophysiology at distinct maturation stages could delineate whether dysregulation of developmental pathways and current differences represent transient maturation lag or persistent alterations. Comparisons to late-onset neurodevelopmental disorders may also contextualize these findings. Overall, caution must be taken in interpreting in vitro disease modeling results, given the inherent developmental components.

In conclusion, our study shows that DA neurons derived from PD patients with E326K-*GBA1* mutations share synaptic deficits similar to other PD mutations. DA neurons from sPD patients present milder synaptic abnormalities compared to other PD mutations. Additionally, several shared pathways, such as ECM–receptor interaction, synaptic pathways, and more, are commonly dysregulated in monogenic and sporadic forms of the disease. These shared changes in the patients’ DA neurons should be considered as possible new targets for drug discovery.

## Methods

### Human patients

The patients with sporadic PD were diagnosed by Dr. Juergen Winkler, and the patients with E326K-*GBA1* PD were diagnosed by Dr. Henry Houlden. A written informed consent was provided by all the participants in the study.

### Ethics

The study was approved by the University of Haifa: IRB 282-22.

### Midbrain DA differentiation

To generate in vitro midbrain DA neurons, we employed a protocol previously developed and reported in our earlier studies^24,99,100^. We differentiated midbrain DA neurons from 3 unrelated (no family connection to the patients) healthy control individuals, 3 PD patients with the E326K mutation in the *GBA1* gene, and 4 sPD patients. Briefly, human iPSCs were dissociated, replated, and allowed to proliferate before initiating differentiation at 50% confluency (day 0). A gradual transition from KSR medium (DMEM F-12 with Glutamax, KO-SR, NEAA, β-mercaptoethanol) to N2 medium (DMEM F-12 with Glutamax, N2 supplement) occurred from day 5 to day 10, followed by a switch to B27 medium (Neurobasal medium, B27 supplement, glutamax, BDNF, GDNF, TGFβ3, ascorbic acid, and cyclic adenosine monophosphate (cAMP)) on day 11. Various small molecules were added during the differentiation, such as SB431542, LDN-193189, Smoothened agonist (SAG), and fibroblast growth factor (FGF) 8b. Neurons were dissociated and replated on days 20-25 and maintained in the B27 medium until day 30 when the base medium was replaced with Brainphys medium^101^ to promote synaptic connections. Whole-cell patch clamp and RNA sequencing experiments were conducted on mature neurons (more than 7 weeks in differentiation).

### Immunocytochemistry (ICC)

Coverslips with DA neurons were fixed in 4% paraformaldehyde (PFA) for 15 min at 37 °C. After washing with DPBS, the cells were blocked and permeabilized in a solution of DPBS, 0.2% Molecular Grade Triton X-100, and 10% Donor Horse Serum for one hour. Primary antibodies were added to the blocking solution at 4 °C overnight, with the following dilutions for DA neurons: TH (Abcam ab129991) (1:500) and MAP2 (Abcam ab92434) (1:500) (please note that while MAP2 is mainly expressed in neurons, there may be some low expression in glial cells). The next day, the coverslips were washed with DPBS and incubated with Alexa Fluor secondary antibodies, followed by counterstaining with DAPI staining solution (1:3000) for one hour at room temperature. The coverslips were rewashed, mounted on slides using Fluoromount-G mounting medium (0100-01, Southern Biotech), and allowed to dry overnight in the dark. The fluorescence signals were visualized using a Nikon A1-R confocal microscope, and images were processed using NIS elements 5.21 (Nikon) and microscopy image analysis software Imaris 9.8 (Oxford Instruments).

### RNA extraction, sequencing, and analyses

The total RNA was extracted from 3 to 5 million DA neurons per sample, derived from three patients with E326K-*GBA1* mutations, two patients with sPD, and three healthy control lines at 7–8 weeks post-differentiation, using the zymo RNA clean & concentrator kit as per the manufacturer’s instructions. Two biological replicates were performed for each of the cell lines. The extracted RNA was reverse transcribed with the high-capacity cDNA synthesis kit from AB Biosystems.

To analyze the RNA sequencing data reads sequenced on a next-generation sequencing (NGS) platform were processed in FASTQ-format files. The sequences were trimmed using the Trimmomatic algorithm and quality-tested using FASTQC v0.11.8. Sequences were aligned to the hg38 human genome using STAR aligner v2.78a. Mapping was carried out using default parameters, filtering non-canonical introns, allowing up to 10 mismatches per read, and only keeping uniquely mapped reads. The expression levels of each gene were quantified by counting the number of reads that aligned to each exon or full-length transcript and normalized by its mean across all samples using HTseq v0.9.1. Differentially expressed genes (DEGs) were determined using DESeq1 v.2.11.40.7, and the *p*-value was adjusted for multiple hypotheses with the Benjamini–Hochberg procedure^102^, which controls for the false discovery rate (FDR). Genes with an FDR < 0.05 and |log2 fold change | > 0.5 were included in the analysis. A Gene Ontology (GO) enrichment test and KEGG pathway analysis were performed using expressAnalyst. A graphical representation of the protein network pathways analysis was constructed using nodes and edges, where each node represented a pathway connected by the edges (lines). The size of each node is proportional to the number of DEGs. An overrepresentation of GO terms and KEGG pathway was determined by FDR < 0.05.

### Patch-clamp recordings of dopaminergic neurons

Whole-cell patch-clamp recordings were performed from DA neuronal cultures. The recordings were conducted at 45–50 days of the differentiation. Each of the patient and control lines were differentiated 2–3 times and the data for each of the lines was obtained from 6 to 7 coverslips. Culture coverslips were placed inside a recording chamber filled with HEPES-based artificial cerebrospinal fluid (ACSF) containing NaCl (139 mM), HEPES (10 mM), KCl (4 mM), CaCl_2_ (2 mM), d-glucose (10 mM), and MgCl2 (1 mM) at pH 7.4 and osmolarity adjusted to 310 mOsm at room temperature. Recording micropipettes with a tip resistance of 10–15 MΩ were filled with an internal solution containing K-gluconate (130 mM), KCl (6 mM), NaCl (4 mM), Na-HEPES (10 mM), K-EGTA (0.2 mM), GTP (0.3 mM), Mg-ATP (2 mM), cAMP (0.2 mM), d-glucose (10 mM), biocytin (0.15%), and rhodamine (0.06%) at a pH of 7.4 and osmolarity adjusted to 290–300 mOsm. Data were recorded at a sampling rate of 20 kHz using Clampex v11.1.

### Analysis of total evoked action potentials

To determine the total number of evoked action potentials, neurons were held in current clamp mode at a steady membrane potential of −60 mV with a constant holding current. Current injections were given in 3 pA steps over 400 ms, starting 12 pA below the steady-hold current required for a −60 mV membrane potential. The total number of evoked action potentials in response to the first 32 depolarization steps within the 400 ms recordings was counted and compared between the groups as a measure of the excitability.

### Input conductance and capacitance

The input conductance was determined by measuring the current while holding the cell in voltage-clamp mode at −70 and −50 mV, respectively, around the resting membrane potential. The input conductance was calculated by dividing the difference in currents by the difference in membrane potentials (20 mV). The capacitance was measured using the membrane test feature in the Clampex software.

### Analysis of sodium, fast, and slow potassium currents

Sodium and potassium current measurements were performed in voltage-clamp mode. Voltage test steps of 400 ms in the range of −90 to 80 mV were produced while holding the cells at a voltage of −60 mV. The measured currents were normalized by the cell capacitance. The fast potassium current was measured as the maximal outgoing current within a few milliseconds after a depolarization step, while the slow potassium current was measured at the end of the 400 ms depolarization step. The amplitudes of the sodium and potassium currents were statistically analyzed to compare PD vs. control DA neurons at specific test potentials (−20 to 0 mV for the sodium current and 50–90 mV for the potassium current).

### Analysis of synaptic activity

Spontaneous excitatory post-synaptic currents (EPSCs) were recorded in a voltage-clamp mode to analyze synaptic activity. Neurons were held at −60 mV, and the amplitude and rate of synaptic activity were evaluated using a custom-written Matlab code. The cumulative distribution of EPSC amplitudes was calculated for each group. The EPSC event rates and amplitudes were calculated as the average of events per each recorded cell.

We additionally calculated the EPSC rate of large EPSCs whose amplitudes were >30 pA.

### Statistical analyses

For the comparison of evoked action potentials, EPSC rates and amplitudes, capacitance, and input conductance, the Mann–Whitney *U* test (a non-parametric test) was employed. This choice was driven by the data characteristics; While some datasets followed a normal distribution, others did not. To ensure consistency across different types of data, the Mann–Whitney *U* test was used as it does not require the assumption of normal distribution.

For the analysis of sodium and potassium current changes at various potentials, a one-way analysis of variance (ANOVA) was utilized. Prior to conducting the ANOVA, tests for normality and homogeneity of variances were performed to ensure the validity of the ANOVA assumptions. Specifically, the Kolmogorov–Smirnov test was used to assess normality, while Levene’s test was employed to evaluate the homogeneity of variances across groups.

All data values are presented as mean ± standard error (SE). A *p*-value of <0.05 was considered significant for all statistical tests.

### Reporting summary

Further information on research design is available in the Nature Research Reporting Summary linked to this article.

### Supplementary information

Supplementary information

Reporting Summary

## Data Availability

The data used in this study are available upon request to the corresponding author. RNA sequencing data is available on the following web address (https://usegalaxy.org/u/yara_hu/h/gba1) and on the NCBI portal under Bioprojects PRJNA1068326 and PRJNA1068496. Please contact Shani Stern at sstern@unive.haifa.ac.il for access to the whole cell patch clamp data.

## Code availability

The data used in this study are available upon request to the corresponding author.
