# Supplementary material for: Synaptic dysfunction and extracellular matrix dysregulation in dopaminergic neurons from sporadic and E326K-GBA1 Parkinson’s disease patients
Source: NPJ Parkinsons Dis. 2024 Feb 19;10:38. doi: 10.1038/s41531-024-00653-x (PMC10876637; doi:10.1038/s41531-024-00653-x)
Supplement: Supplementary file 1 — Supplementary information [file 41531_2024_653_MOESM1_ESM.pdf]

Supplementary Figure Legends

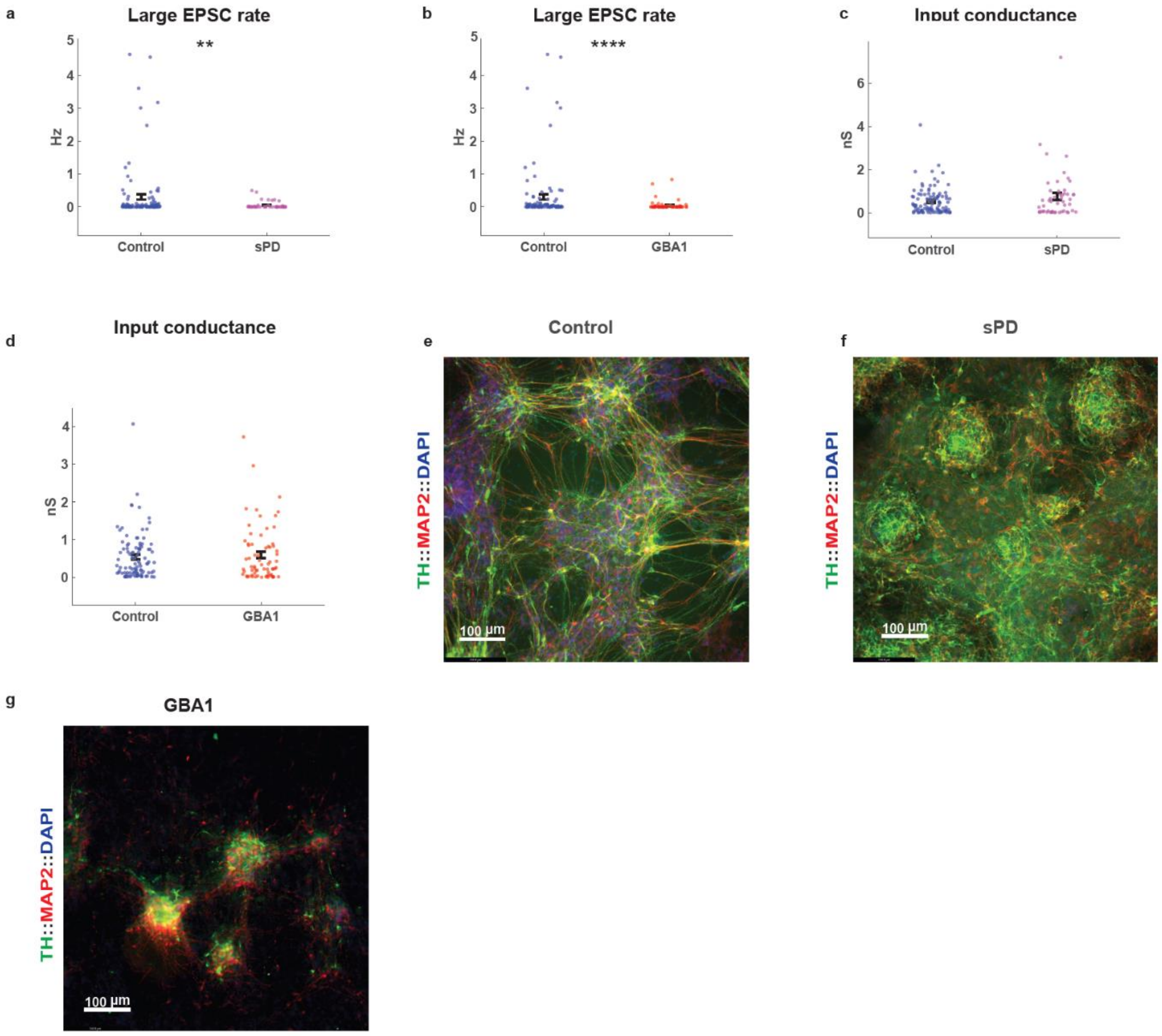

Supplementary Figure 1

(a) The large EPSC rate was significantly decreased in sPD neurons compared to control neurons. (b) The large EPSC rate was also significantly decreased in E326K-*GBA1* neurons compared to control neurons. (c) The input conductance was not significantly different between control and sPD neurons. (d) The input conductance was not significantly different between control and E326K-*GBA1* neurons. An example image

of DA neurons with ICC for Tyrosine Hydroxylase (TH) (green), MAP2 (red), DAPI (blue) in a (e) control (f) sPD, and (g) E326K-*GBA1* DA neuronal cultures.

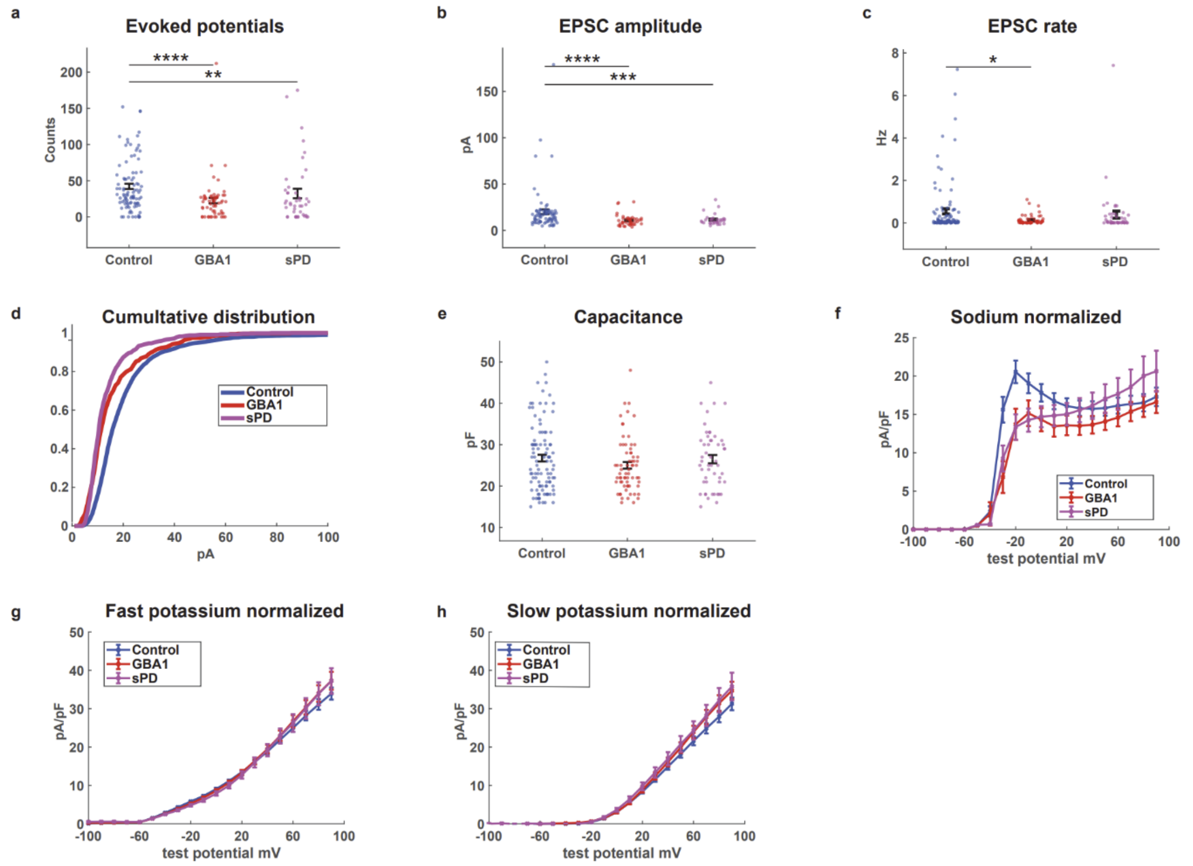

Supplementary Figure 2

This figure shows a comparison between Control, E326K-*GBA1*, and sPD neurons together. (a) Both E326K-*GBA1* and sPD neurons were hypoexcitable compared to control neurons. (b) The EPSC amplitude was decreased in both E326K-*GBA1* and sPD compared to control neurons. (c) The EPSC rate in E326K-*GBA1* neurons was significantly reduced compared to control neurons (d) The cumulative distribution of EPSC amplitudes was left shited in both sPD and E326K-*GBA1* neurons indicating smaller amplitudes. (e) The capacitance was similar between all groups. (f) The sodium currents were reduced in both E326K-*GBA1* and sPD

neurons compared to control neurons. (g) The fast potassium and (h) slow potassium currents were not significantly different between E326K-*GBA1*, sPD, and control neurons.

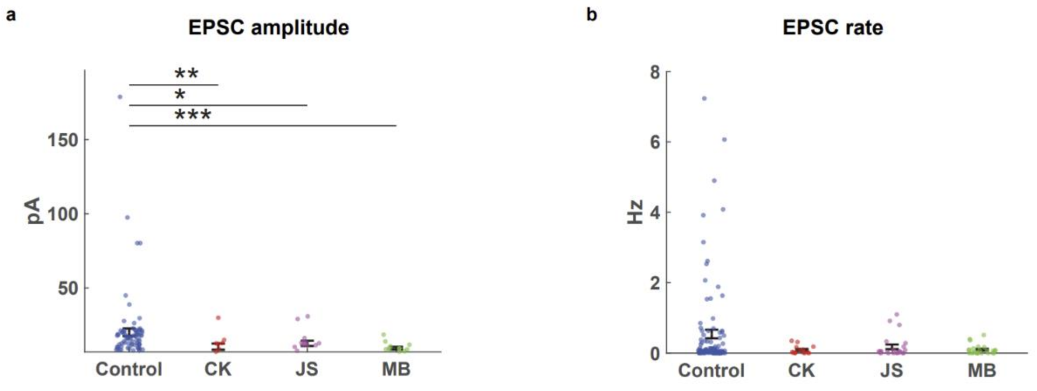

Supp. Figure 3

This figure shows comparisons among individual E326K-*GBA1* patients compared to the control group. (a) The EPSC amplitude was significantly decreased in all E326K-*GBA1* lines compared to the controls. (b) The EPSC rate in all E326K-*GBA1* lines showed a trend of a decreased mean value compared to controls (but not significantly).

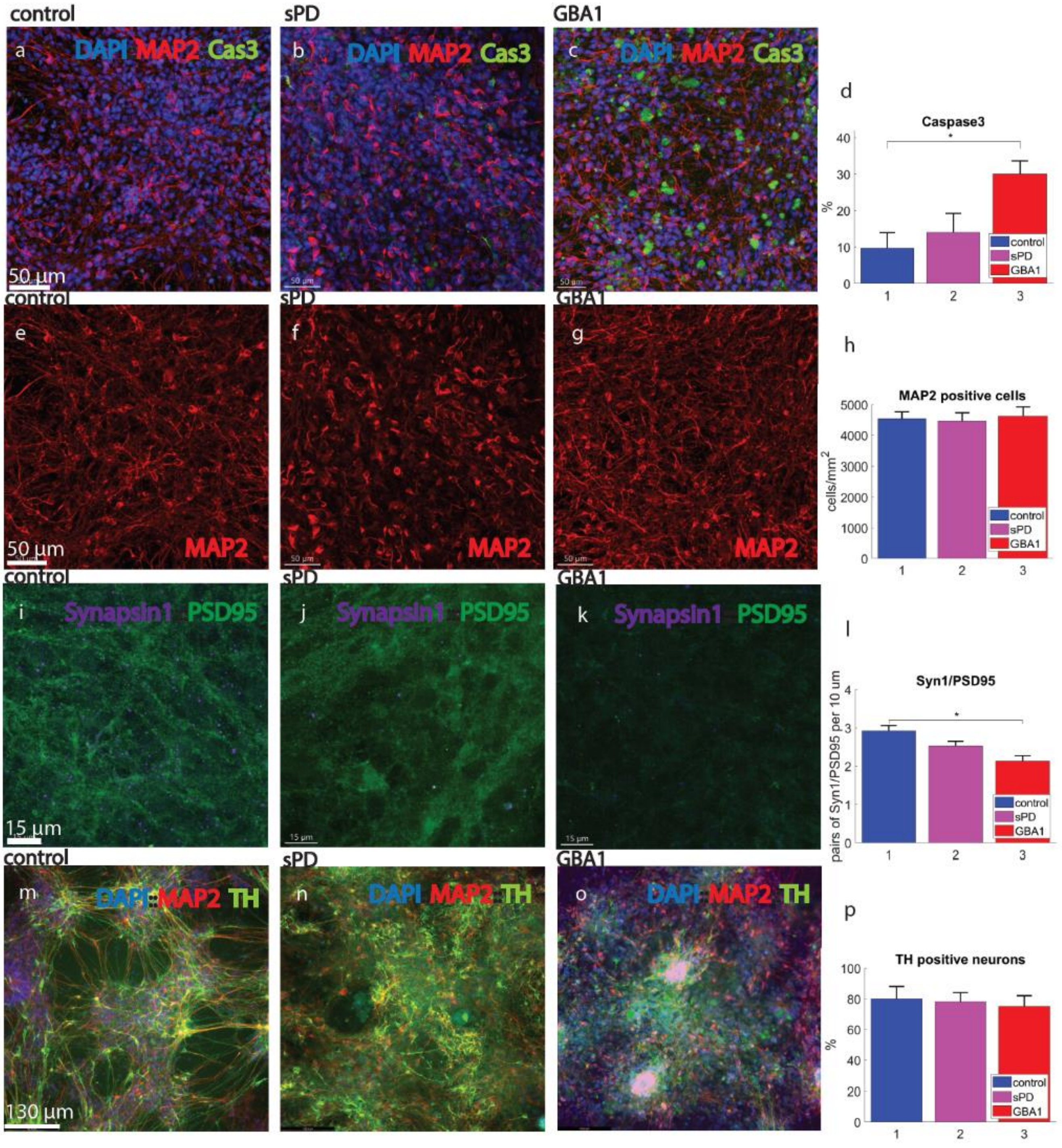

Supplementary Figure 4, ICC

(a-d) Caspase3 protein expression is elevated in E326K-*GBA1* neuronal cultures. Representative traces (a-c) and the averages (d) are shown. (e-h) Despite more Caspase3 positive cells in the E326K-*GBA1* cultures, the density of MAP2 positive neurons was the same between the three groups. Representative images (e-g) and the averages (h) are presented. (i-l)

A reduction in the density of Synapsin1::PSD95 pairs was observed in the E326K-*GBA1* neuronal cultures. Representative images (i-k) and the averages (l) are presented. (m-p) The percentage of TH positive neurons was more than 80% on average in all the three groups. Representative images (m-o) and the averages (p) are presented.

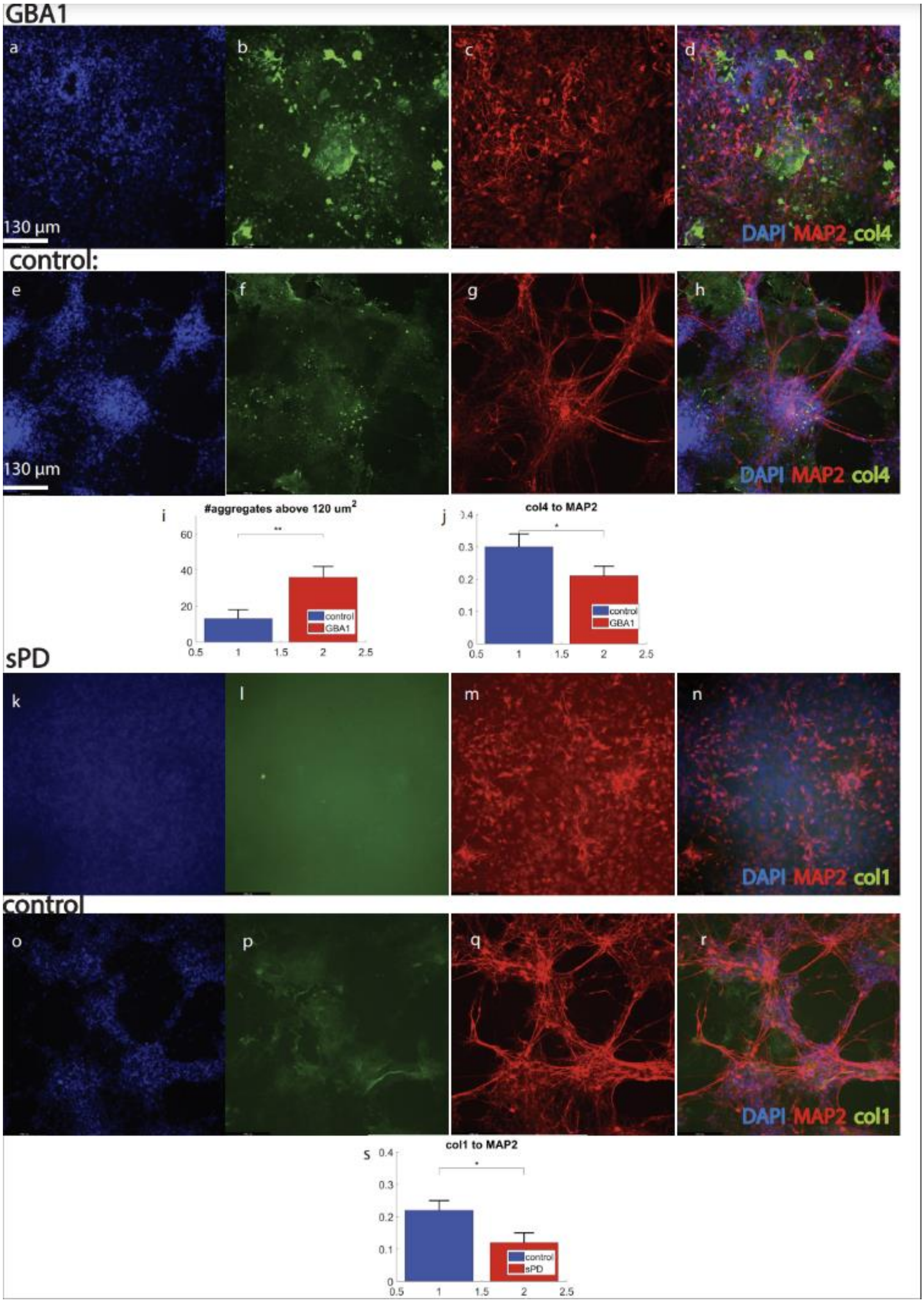

Supplementary Figure 5, ICC

ICC images for DAPI (blue), MAP2(red), and collagen 4 (green) in the 2 upper rows and collagen1 (green) in the 2 bottom rows. (a-d) Example ICC images for a E326K-*GBA1* culture. (e-h) Example ICC images for a control culture. (i) E326K-*GBA1* DA neuronal cultures exhibited more aggregation of the collagen 4 protein compared to control DA neurons culture. (j) However, the diffuse background of collagen 4 in the E326K-*GBA1* DA neuronal cultures had a lower intensity compared to control DA neuronal cultures. (k-n) Example ICC images for an sPD culture. (o-r) Example ICC images for a control culture. (s) The diffuse background of collagen 1 in the sPD DA neuronal cultures had a lower intensity compared to control DA neuronal cultures.

Supplementary tables

Enrichment_kegg_GBA1_vs_Control					
Pathway	Total	Expected	Hits	P.Value	FDR
Basal cell carcinoma	63	2.28	10	7.67E-05	0.0176
ECM-receptor interaction	82	2.97	11	0.000163	0.0176
Pathways in cancer	530	19.2	36	0.000166	0.0176
Hypertrophic cardiomyopathy (HCM)	85	3.08	11	0.000226	0.0179
Focal adhesion	199	7.2	17	0.00085	0.0541
Arrhythmogenic right ventricular cardiomyopathy (ARVC)	72	2.61	9	0.00108	0.0571
Hippo signaling pathway	154	5.57	14	0.00133	0.0605
Dilated cardiomyopathy	91	3.29	10	0.00158	0.0629
Phenylalanine metabolism	17	0.615	4	0.00275	0.0972
Sulfur metabolism	9	0.326	3	0.00335	0.106
Signaling pathways regulating pluripotency of stem cells	139	5.03	12	0.00442	0.114
PI3K-Akt signaling pathway	354	12.8	23	0.00455	0.114
Regulation of actin cytoskeleton	214	7.74	16	0.00464	0.114
Breast cancer	147	5.32	12	0.00689	0.156
Mannose type O-glycan biosynthesis	23	0.832	4	0.00863	0.183
Wnt signaling pathway	158	5.72	12	0.0119	0.237
Melanoma	72	2.61	7	0.0149	0.279
Protein digestion and absorption	90	3.26	8	0.0159	0.28
Transcriptional misregulation in cancer	186	6.73	13	0.0173	0.289
FoxO signaling pathway	132	4.78	10	0.0211	0.336
AGE-RAGE signaling pathway in diabetic complications	100	3.62	8	0.028	0.425
Proteoglycans in cancer	201	7.27	13	0.0304	0.439
Arginine and proline metabolism	50	1.81	5	0.0337	0.446
N-Glycan biosynthesis	50	1.81	5	0.0337	0.446
Neuroactive ligand-receptor interaction	338	12.2	19	0.0372	0.465
Adipocytokine signaling pathway	69	2.5	6	0.0381	0.465
Insulin resistance	108	3.91	8	0.0416	0.477
Other types of O-glycan biosynthesis	22	0.796	3	0.0434	0.477
African trypanosomiasis	37	1.34	4	0.0435	0.477
Purine metabolism	130	4.7	9	0.0458	0.483
TGF-beta signaling pathway	92	3.33	7	0.0486	0.483
Proximal tubule bicarbonate reclamation	23	0.832	3	0.0487	0.483

Supplementary Table 1. KEGG pathway enrichment analysis of differentially expressed genes (DEGs) in E326K-*GBA1* versus control neurons. The table lists significantly enriched pathways, the number of genes in each pathway, the expected number of DEGs in the pathway, the actual number of DEGs observed in the pathway, p-values from the performed enrichment test, and Benjamini-Hochberg adjusted FDR p-values (q-values). Pathways are ordered by statistical significance.

enrichment_kegg_GBA1_downregulated_vs_Control					
Pathway	Total	Expected	Hits	P.Value	FDR
Pathways in cancer	530	13.2	29	4.14E-05	0.0132
Focal adhesion	199	4.94	15	0.000119	0.0189
PI3K-Akt signaling pathway	354	8.78	20	0.000471	0.0488
Jak-STAT signaling pathway	162	4.02	12	0.000673	0.0488
Regulation of actin cytoskeleton	214	5.31	14	0.000848	0.0488
ECM-receptor interaction	82	2.03	8	0.000921	0.0488
Hypertrophic cardiomyopathy (HCM)	85	2.11	8	0.00117	0.0531
Arrhythmogenic right ventricular cardiomyopathy (ARVC)	72	1.79	7	0.00196	0.0779
Proteoglycans in cancer	201	4.99	12	0.0042	0.144
Th17 cell differentiation	107	2.66	8	0.00498	0.144
Insulin resistance	108	2.68	8	0.00527	0.144
FoxO signaling pathway	132	3.28	9	0.00544	0.144
Dilated cardiomyopathy	91	2.26	7	0.00726	0.178
Melanoma	72	1.79	6	0.00868	0.197
AGE-RAGE signaling pathway in diabetic complications	100	2.48	7	0.0119	0.237
African trypanosomiasis	37	0.918	4	0.0128	0.237
EGFR tyrosine kinase inhibitor resistance	79	1.96	6	0.0134	0.237
Complement and coagulation cascades	79	1.96	6	0.0134	0.237
Transcriptional misregulation in cancer	186	4.62	10	0.0172	0.288
Basal cell carcinoma	63	1.56	5	0.0196	0.312
Adipocytokine signaling pathway	69	1.71	5	0.0279	0.422
Prolactin signaling pathway	70	1.74	5	0.0295	0.422
Malaria	49	1.22	4	0.0327	0.422
Adherens junction	72	1.79	5	0.0328	0.422
Hematopoietic cell lineage	97	2.41	6	0.0332	0.422
Bacterial invasion of epithelial cells	74	1.84	5	0.0363	0.444
Pancreatic cancer	75	1.86	5	0.0381	0.449
Pertussis	76	1.89	5	0.04	0.454
Kaposi's sarcoma-associated herpesvirus infection	186	4.62	9	0.0417	0.457

Supplementary Table 2. Downregulated and upregulated (separately) KEGG pathways in E326K-*GBA1* versus control neurons. Significantly enriched pathways for downregulated DEGs, with stats and enrichment analysis results displayed as in Supplementary Table 1.

enrichment_go_bp_GBA1					
Pathway	Total	Expected	Hits	P.Value	FDR
Muscle organ development	389	13.7	34	1.07E-06	0.000878
Skeletal muscle tissue development	197	6.95	21	6.43E-06	0.00263
Regulation of anatomical structure morphogenesis	702	24.8	47	1.79E-05	0.00398
Positive regulation of epithelial cell proliferation	120	4.23	15	2.07E-05	0.00398
Gland development	303	10.7	26	2.75E-05	0.00398
Negative regulation of developmental process	674	23.8	45	2.91E-05	0.00398
Negative regulation of cell differentiation	540	19	38	4.06E-05	0.00476
Tissue morphogenesis	566	20	39	5.10E-05	0.00523
Central nervous system development	784	27.7	49	6.92E-05	0.0063
Morphogenesis of an epithelium	440	15.5	32	8.97E-05	0.00735
Positive regulation of transcription from RNA polymerase II promoter	800	28.2	49	0.000114	0.00823
Vasculature development	652	23	42	0.00012	0.00823
Tissue remodeling	126	4.44	14	0.000142	0.00856
Striated muscle tissue development	354	12.5	27	0.000146	0.00856
Regulation of cell differentiation	1290	45.5	70	0.00017	0.00927
Skeletal system development	459	16.2	32	0.000195	0.00942
Cell development	1840	65	93	0.000195	0.00942
Regulation of developmental process	1880	66.2	94	0.000234	0.0107
Negative regulation of response to stimulus	967	34.1	55	0.000292	0.0126
Anatomical structure formation involved in morphogenesis	2090	73.7	102	0.000308	0.0126
Regulation of sequence_specific DNA binding transcription factor activity	372	13.1	27	0.000325	0.0126
Growth	839	29.6	49	0.000348	0.0126
Brain development	559	19.7	36	0.00037	0.0126
Endothelial cell proliferation	92	3.25	11	0.000384	0.0126
Negative regulation of transferase activity	189	6.67	17	0.000385	0.0126
Regulation of endothelial cell proliferation	79	2.79	10	0.000442	0.0139
Organ morphogenesis	966	34.1	54	0.000504	0.0148
Positive regulation of cell proliferation	786	27.7	46	0.000506	0.0148
Developmental growth	290	10.2	22	0.000633	0.0179
Cell fate commitment	254	8.96	20	0.000694	0.019
Positive regulation of peptidyl_tyrosine phosphorylation	134	4.73	13	0.000906	0.0239
Tissue development	1680	59.4	83	0.000942	0.0239
Tube development	506	17.8	32	0.00105	0.0239
Sensory organ development	485	17.1	31	0.00105	0.0239
Positive regulation of cell migration	263	9.28	20	0.00107	0.0239
Positive regulation of signal transduction	998	35.2	54	0.00107	0.0239
Regulation of peptidyl_tyrosine phosphorylation	171	6.03	15	0.00108	0.0239
Negative regulation of sequence_specific DNA binding transcription factor activity	121	4.27	12	0.00116	0.0251
Neurogenesis	1390	49.1	70	0.00142	0.0291
Positive regulation of lymphocyte activation	250	8.82	19	0.00142	0.0291
Cell morphogenesis involved in differentiation	827	29.2	46	0.00146	0.0291
Generation of neurons	1300	46	66	0.0017	0.0331
Neuron differentiation	1190	41.8	61	0.00176	0.0335

Positive regulation of binding	79	2.79	9	0.00181	0.0337
Positive regulation of cell differentiation	571	20.1	34	0.00204	0.0371
Regulation of lymphocyte activation	360	12.7	24	0.00218	0.0384
Regulation of neurogenesis	444	15.7	28	0.0022	0.0384
Embryonic morphogenesis	581	20.5	34	0.00269	0.0453
Regulation of cell morphogenesis	325	11.5	22	0.00272	0.0453
Regulation of cell proliferation	1430	50.4	70	0.00276	0.0453
Tube morphogenesis	347	12.2	23	0.00287	0.0456
Regulation of binding	189	6.67	15	0.00289	0.0456
Negative regulation of cell proliferation	585	20.6	34	0.003	0.0464
Negative regulation of signal transduction	790	27.9	43	0.00309	0.0469
Regulation of cell migration	456	16.1	28	0.00321	0.0479
Nervous system development	2190	77.4	100	0.00341	0.0494
Regulation of T cell proliferation	120	4.23	11	0.00343	0.0494
Pattern specification process	503	17.7	30	0.00357	0.0505
Neuron development	945	33.3	49	0.00425	0.0584
Negative regulation of growth	197	6.95	15	0.00427	0.0584
Angiogenesis	426	15	26	0.0048	0.0645
Cell_substrate adhesion	241	8.5	17	0.00529	0.07
Positive regulation of developmental process	817	28.8	43	0.0056	0.0729
Hematopoietic or lymphoid organ development	679	24	37	0.00577	0.074
Glycoprotein metabolic process	458	16.2	27	0.00646	0.0789
Development of primary sexual characteristics	246	8.68	17	0.00647	0.0789
Bone remodeling	63	2.22	7	0.00654	0.0789
Glycoprotein biosynthetic process	393	13.9	24	0.00655	0.0789
Hemopoiesis	640	22.6	35	0.00679	0.0806
Regulation of response to external stimulus	461	16.3	27	0.00703	0.0813
Peptidyl_tyrosine phosphorylation	228	8.04	16	0.00704	0.0813
Regionalization	353	12.5	22	0.00718	0.0818
Peptidyl_tyrosine modification	230	8.11	16	0.00763	0.0857
Cell migration	1050	37.2	52	0.00856	0.0949
B cell activation	194	6.84	14	0.00895	0.0966
Immune system development	722	25.5	38	0.00895	0.0966
Regulation of T cell activation	279	9.84	18	0.0102	0.109
Cell_matrix adhesion	159	5.61	12	0.0107	0.111
Protein processing	140	4.94	11	0.0107	0.111
Positive regulation of transport	570	20.1	31	0.0113	0.116
Regulation of growth	548	19.3	30	0.0116	0.117
Anatomical structure morphogenesis	2820	99.3	120	0.0119	0.119
Regulation of cell shape	106	3.74	9	0.0126	0.124
Peptidyl_amino acid modification	739	26.1	38	0.0127	0.124
Hormone metabolic process	203	7.16	14	0.013	0.125
Embryo development	1080	38.2	52	0.0142	0.136
Response to external stimulus	1510	53.3	69	0.0145	0.136
Regulation of intracellular transport	290	10.2	18	0.0147	0.136
T cell proliferation	147	5.19	11	0.0151	0.136
Regulation of endocytosis	128	4.52	10	0.0152	0.136
Negative regulation of immune system process	207	7.3	14	0.0152	0.136

Organ development	3290	116	137	0.0153	0.136
Positive regulation of T cell proliferation	75	2.65	7	0.0164	0.143
Cell proliferation	1900	67.1	84	0.0164	0.143
Leukocyte differentiation	404	14.3	23	0.0167	0.143
Regulation of angiogenesis	169	5.96	12	0.0167	0.143
Negative regulation of hydrolase activity	253	8.92	16	0.0177	0.149
Negative regulation of transport	318	11.2	19	0.0179	0.149
Positive regulation of cell cycle	113	3.99	9	0.0184	0.149
Regulation of signal transduction	2440	85.9	104	0.0186	0.149
Protein autophosphorylation	192	6.77	13	0.0187	0.149
Myoblast differentiation	44	1.55	5	0.0187	0.149
Glucan metabolic process	95	3.35	8	0.0189	0.149
Regulation of nucleocytoplasmic transport	172	6.07	12	0.0189	0.149
Protein maturation	153	5.4	11	0.0197	0.154
Positive regulation of phosphate metabolic process	789	27.8	39	0.0208	0.161
Regulation of homeostatic process	324	11.4	19	0.0213	0.163
Neuron projection development	816	28.8	40	0.0218	0.166
Gliogenesis	176	6.21	12	0.0222	0.167
Acute inflammatory response	118	4.16	9	0.0237	0.177
Extracellular structure organization	242	8.54	15	0.0249	0.184
Rhythmic process	200	7.05	13	0.0252	0.184
Positive regulation of protein phosphorylation	681	24	34	0.0261	0.19
Enzyme linked receptor protein signaling pathway	1180	41.4	54	0.0265	0.191
Positive regulation of T cell activation	202	7.13	13	0.027	0.193
JAK_STAT cascade	121	4.27	9	0.0274	0.193
Response to steroid hormone stimulus	267	9.42	16	0.0276	0.194
Negative regulation of phosphorylation	246	8.68	15	0.0282	0.196
Regulation of multicellular organismal process	2480	87.4	104	0.0292	0.199
"Positive regulation of transcription DNA_dependent"	1260	44.4	57	0.0293	0.199

Supplementary Table 3. Gene Ontology Biological Process enrichment analysis of DEGs in E326K-*GBA1* versus control neurons. The table displays significantly enriched terms with stats and enrichment analysis results displayed as in Supplementary Table 1.

enrichment_go_cc_GBA1					
Pathway	Total	Expected	Hits	P.Value	FDR
Extracellular region part	1320	42.9	75	1.31E-06	0.000294
Proteinaceous extracellular matrix	398	12.9	31	6.85E-06	0.000771
Extracellular space	901	29.3	54	1.04E-05	0.000782
Receptor complex	189	6.15	18	4.63E-05	0.0026
Extracellular matrix part	204	6.63	17	0.000374	0.0168
Extracellular matrix	570	18.5	34	0.000511	0.0191
Endoplasmic reticulum lumen	175	5.69	15	0.000606	0.0195
Cell surface	518	16.8	31	0.000845	0.0218
Collagen	93	3.02	10	0.000873	0.0218
Basement membrane	100	3.25	10	0.00153	0.0344
Basolateral plasma membrane	162	5.27	13	0.00247	0.0506
Integral to plasma membrane	1270	41.3	59	0.00343	0.0558
Intrinsic to plasma membrane	1320	43	61	0.00347	0.0558
Integrin complex	32	1.04	5	0.00347	0.0558
Integral to Golgi membrane	47	1.53	6	0.00398	0.0597
Intrinsic to Golgi membrane	50	1.63	6	0.00543	0.0763
Extracellular region	2860	92.9	113	0.0131	0.173
Acetylcholine_gated channel complex	17	0.553	3	0.0165	0.207
Actin cytoskeleton	430	14	22	0.0247	0.293
Secretory granule	276	8.97	15	0.0367	0.401
Cytoplasmic vesicle	1110	36.1	47	0.0374	0.401
Vesicle	1210	39.3	50	0.0467	0.478

Supplementary Table 4. Gene Ontology Cellular Component enrichment analysis of DEGs in E326K-*GBA1* versus controls. The table displays significantly enriched terms with stats and enrichment analysis results displayed as in Supplementary Table 1.

enrichment_go_mf_GBA1					
Pathway	Total	Expected	Hits	P.Value	FDR
Transferase activity, transferring hexosyl groups	237	8.31	19	0.000707	0.192
Transferase activity, transferring glycosyl groups	333	11.7	23	0.00159	0.192
Heparin binding	130	4.56	12	0.00206	0.192
Pattern binding	17	0.596	4	0.00247	0.192
Polysaccharide binding	17	0.596	4	0.00247	0.192
Glycosaminoglycan binding	178	6.24	14	0.00406	0.263
RNA polymerase II distal enhancer sequence_specific DNA binding transcription factor activity	110	3.86	10	0.00527	0.292
Growth factor activity	170	5.96	13	0.00696	0.338
Metalloendopeptidase activity	123	4.31	10	0.0113	0.487
Double_stranded DNA binding	149	5.23	11	0.0159	0.565
G_protein coupled receptor binding	232	8.14	15	0.017	0.565
Calmodulin binding	171	6	12	0.0175	0.565
UDP_glycosyltransferase activity	153	5.37	11	0.019	0.568
Acetylglucosaminyltransferase activity	47	1.65	5	0.0238	0.66
Positive regulation of transcription, DNA_dependent	1260	44.1	57	0.0268	0.692
Acetylcholine_activated cation_selective channel activity	21	0.737	3	0.0357	0.865
GTPase activity	241	8.45	14	0.0451	0.954
Structure_specific DNA binding	242	8.49	14	0.0464	0.954
Cytokine activity	220	7.72	13	0.0467	0.954

Supplementary Table 5. Gene Ontology Molecular Function enrichment analysis of DEGs in E326K-*GBA1* versus controls. The table displays significantly enriched terms with stats and enrichment analysis results displayed as in Supplementary Table 1.

enrichment_GO_CC_sPD				
Pathway	Total	Hits	Enrichment	FDR
Neuron projection	1444	71	2.259790233	5.29E-08
External encapsulating structure	601	39	2.982408352	2.12E-07
Extracellular matrix	600	39	2.987379032	2.12E-07
Synapse	1443	68	2.165806005	2.12E-07
Collagen-containing extracellular matrix	446	32	3.297555331	4.87E-07
Receptor complex	422	28	3.04945727	1.95E-05
Plasma membrane region	1323	58	2.014861142	2.52E-05
Integral component of plasma membrane	1881	74	1.808089383	3.22E-05
Intrinsic component of plasma membrane	1965	76	1.77757531	3.78E-05
Synaptic membrane	401	26	2.979929209	4.94E-05
Postsynapse	682	36	2.426024028	5.49E-05
Somatodendritic compartment	913	43	2.164585026	9.18E-05
Postsynaptic membrane	297	21	3.249674161	0.000112
Plasma membrane signaling receptor complex	194	16	3.790488859	0.000237
Postsynaptic specialization membrane	121	12	4.557984537	0.000514
Fibrillar collagen trimer	16	5	14.36239919	0.000544
AMPA glutamate receptor complex	26	6	10.6060794	0.000544
Banded collagen fibril	16	5	14.36239919	0.000544
Synaptic vesicle	213	16	3.452370135	0.000564
Neuronal cell body	535	28	2.405366295	0.000564
Axon	691	33	2.194890528	0.000641
Postsynaptic specialization	376	22	2.689130062	0.000767
Integral component of postsynaptic specialization membrane	74	9	5.589690497	0.000797
Postsynaptic density	352	21	2.741912573	0.000801
Synaptic vesicle membrane	114	11	4.434705716	0.000819
Asymmetric synapse	358	21	2.695958731	0.000819
Cell body	614	30	2.245586845	0.000819
Glutamatergic synapse	358	21	2.695958731	0.000819
Neuron to neuron synapse	385	22	2.626267281	0.000819
Exocytic vesicle membrane	114	11	4.434705716	0.000819
Intrinsic component of postsynaptic specialization membrane	78	9	5.303039702	0.000904
Exocytic vesicle	234	16	3.142542046	0.001051
Dendrite	674	31	2.113872404	0.001433
Excitatory synapse	49	7	6.565668203	0.001433
Dendritic tree	676	31	2.107618343	0.001433
Complex of collagen trimers	25	5	9.191935484	0.002814
Postsynaptic density membrane	95	9	4.354074703	0.003523
Presynapse	527	25	2.180250352	0.003789
Dendrite membrane	43	6	6.412978245	0.004649
Secretory vesicle	1150	43	1.718492286	0.005866
Collagen trimer	105	9	3.939400922	0.006406
Neuron projection membrane	64	7	5.026839718	0.006406
Plasma membrane protein complex	712	30	1.936503262	0.006406
Ionotropic glutamate receptor complex	47	6	5.867192862	0.006745

Synaptic cleft	18	4	10.21326165	0.006758
Neurotransmitter receptor complex	49	6	5.627715602	0.0081
Integral component of postsynaptic density membrane	51	6	5.407020873	0.009843
Dendritic spine	193	12	2.857596523	0.013227
GABA-ergic synapse	74	7	4.347537053	0.013227
Golgi apparatus	1812	59	1.496479563	0.013477
Cell projection membrane	368	18	2.2480277	0.013477
Neuron spine	196	12	2.813857801	0.013477
Integral component of postsynaptic membrane	119	9	3.47594199	0.013477
Intrinsic component of postsynaptic density membrane	55	6	5.013782991	0.013477
Intrinsic component of postsynaptic membrane	125	9	3.309096774	0.01742
Endoplasmic reticulum lumen	328	16	2.241935484	0.024315
Basolateral plasma membrane	240	13	2.489482527	0.024382
Parallel fiber to Purkinje cell synapse	13	3	10.6060794	0.024544
Collagen type V trimer	4	2	22.97983871	0.026696
Leading edge membrane	189	11	2.67490186	0.028219
Basal part of cell	283	14	2.273623618	0.035371
G protein-coupled receptor complex	15	3	9.191935484	0.035371
Calcitonin family receptor complex	5	2	18.38387097	0.041069
Neuron projection terminus	147	9	2.813857801	0.044432
Ion channel complex	324	15	2.127762843	0.045651
Basement membrane	97	7	3.316677752	0.045814
Catenin complex	33	4	5.57086999	0.046928
Basal plasma membrane	265	13	2.254625685	0.047111

Supplementary Table 6. Gene Ontology Cellular Component enrichment analysis of DEGs in sporadic versus controls. The table lists significantly enriched pathways, the number of genes in each pathway, the actual number of DEGs in the pathway, fold enrichment, and the Benjamini-Hochberg adjusted FDR p-values (q-values). Pathways are ordered by statistical significance.

enrichment_GO_upregulated_CC_sPD				
Pathway	Total	Hits	Enrichment	FDR
Collagen-containing extracellular matrix	446	18	3.739910314	0.000838
External encapsulating structure	601	20	3.083749307	0.001366
Extracellular matrix	600	20	3.088888889	0.001366
Plasma membrane region	1323	28	1.961199295	0.057905
Synaptic vesicle	213	8	3.480438185	0.111835
Neuron projection	1444	28	1.796860572	0.111835

Supplementary Table 7. Upregulated Gene Ontology Cellular Component in sporadic versus controls. Enriched Cellular Components for upregulated genes with layout as Supplementary Table 6.

enrichment_kegg_sPD				
Pathway	Total	Hits	Enrichment	FDR
Steroid biosynthesis	20	6	13.7879	0.000859
Protein digestion and absorption	103	10	4.462105	0.011724
Neuroactive ligand-receptor interaction	350	19	2.494954	0.018706
Arrhythmogenic right ventricular cardiomyopathy	77	8	4.775031	0.018706
Metabolic pathways	1527	53	1.595195	0.029583
Hypertrophic cardiomyopathy	90	8	4.085305	0.035943
Nicotinate and nicotinamide metabolism	35	5	6.565668	0.036069
Terpenoid backbone biosynthesis	22	4	8.356305	0.039046
AMPK signaling pathway	120	9	3.446976	0.039046
Dilated cardiomyopathy	96	7	3.351226	0.139387

Supplementary Table 8. KEGG pathway enrichment analysis of DEGs showing dysregulated pathways in sporadic versus controls. Enriched Cellular Components for upregulated genes with layout as Supplementary Table 6.

enrichment_GO_BP_sPD				
Pathway	Total	Hits	Enrichment	FDR
Cholesterol biosynthetic process	59	13	10.12670858	8.99E-07
Secondary alcohol biosynthetic process	59	13	10.12670858	8.99E-07
Sterol biosynthetic process	66	13	9.052663734	2.64E-06
Circulatory system development	1127	55	2.242930131	2.59E-05
Cell adhesion	1639	70	1.962890433	4.16E-05
Biological adhesion	1646	70	1.954542782	4.16E-05
Extracellular matrix organization	450	30	3.063978495	4.16E-05
Extracellular structure organization	451	30	3.057184751	4.16E-05
Supramolecular fiber organization	919	47	2.350494928	4.16E-05
External encapsulating structure organization	454	30	3.036983089	4.33E-05
Central nervous system development	1105	52	2.162808349	6.85E-05
Generation of neurons	1624	68	1.92441999	6.85E-05
Lipid biosynthetic process	838	43	2.358312803	8.08E-05
Export from cell	1545	65	1.933578662	9.24E-05
Regulation of neurotransmitter receptor activity	88	12	6.267228739	0.000159
Anatomical structure formation involved in morphogenesis	1183	53	2.059055709	0.000179
Alcohol biosynthetic process	145	15	4.754449388	0.000197
Organic hydroxy compound biosynthetic process	250	20	3.676774194	0.000197
Cholesterol metabolic process	146	15	4.721884666	0.000204
Secretion	1636	66	1.854119016	0.000226
Head development	856	42	2.255030901	0.000226
Sterol metabolic process	169	16	4.351212063	0.000228
Neuron differentiation	1473	61	1.90328603	0.000232
Neurogenesis	1757	69	1.804904805	0.000273
Brain development	811	40	2.266815162	0.000295
Secretion by cell	1489	61	1.882834333	0.000295
Locomotion	1982	75	1.739140165	0.000317
Secondary alcohol metabolic process	156	15	4.419199752	0.000322
Collagen fibril organization	121	13	4.937816582	0.000441
Lipid metabolic process	1555	62	1.832475884	0.000498
Regulation of transport	1910	72	1.7325114	0.000528
Vasculature development	748	37	2.273406503	0.000562
Forebrain development	411	25	2.795600816	0.000699
Regulation of secretion by cell	611	32	2.407053482	0.000773
Cellular lipid metabolic process	1178	50	1.950750315	0.000799
Response to endogenous stimulus	1769	67	1.740700049	0.000898
Response to organonitrogen compound	1121	48	1.967943369	0.000922
Regulation of locomotion	1056	46	2.002031403	0.000922
Cell-cell signaling	1885	70	1.706725421	0.000953
Regulation of cellular component movement	1092	47	1.978117984	0.000953
Tube development	1094	47	1.974501681	0.00097
Trans-synaptic signaling	776	37	2.191376372	0.000981
Blood vessel development	717	35	2.243498898	0.001027
Neuron development	1203	50	1.910211032	0.001124
Steroid biosynthetic process	202	16	3.640370489	0.001137

Cell motility	1776	66	1.707960985	0.001496
Localization of cell	1776	66	1.707960985	0.001496
Chemical synaptic transmission	767	36	2.157168692	0.001537
Anterograde trans-synaptic signaling	767	36	2.157168692	0.001537
Regulation of plasma membrane bounded cell projection organization	644	32	2.283710679	0.001537
Response to nitrogen compound	1231	50	1.866761877	0.001788
Response to organic cyclic compound	1034	44	1.955730954	0.001874
Small molecule biosynthetic process	623	31	2.286918138	0.001874
Positive regulation of developmental process	1373	54	1.8075911	0.001874
Muscle structure development	627	31	2.272328549	0.002071
Synaptic signaling	815	37	2.086512963	0.002147
Regulation of cell motility	1010	43	1.956699138	0.002147
Regulation of cell projection organization	661	32	2.224976819	0.002188
Regulation of cation channel activity	197	15	3.499467824	0.002603
Regulation of secretion	670	32	2.195089071	0.002749
Response to oxygen-containing compound	1832	66	1.655752571	0.003029
Cholesterol biosynthetic process via desmosterol	4	3	34.46975806	0.003378
Cholesterol biosynthetic process via lathosterol	4	3	34.46975806	0.003378
Hindbrain development	160	13	3.73422379	0.004139
Regulation of ion transmembrane transporter activity	282	18	2.933596431	0.004139
Cellular component morphogenesis	815	36	2.03012072	0.004139
Tube morphogenesis	886	38	1.971182553	0.004717
Organophosphate metabolic process	1121	45	1.844946908	0.004743
Muscle organ development	340	20	2.703510436	0.004785
Regulation of neuron projection development	453	24	2.434949797	0.004785
Blood vessel morphogenesis	635	30	2.171323343	0.004987
Fluid transport	33	6	8.356304985	0.005076
Regulation of system process	606	29	2.199390504	0.005116
Animal organ morphogenesis	1133	45	1.825406429	0.005506
Cell migration	1590	58	1.676516535	0.005506
Regulation of transmembrane transporter activity	293	18	2.823461411	0.005795
Heart development	583	28	2.207325845	0.006066
Cartilage condensation	22	5	10.44538123	0.006273
Neuron projection development	1045	42	1.847183207	0.00703
Ion transport	1720	61	1.629965304	0.007196
Regulation of cell migration	946	39	1.894743572	0.007196
Water transport	23	5	9.991234222	0.007398
Telencephalon development	274	17	2.851512833	0.007398
Regulation of ion transmembrane transport	533	26	2.241935484	0.007708
Intestinal epithelial cell development	13	4	14.14143921	0.008415
Regulation of transporter activity	306	18	2.703510436	0.008841
Regulation of membrane potential	451	23	2.343841642	0.009447
Regulation of ion transport	763	33	1.987771107	0.009447
Regulation of lipid metabolic process	394	21	2.449627477	0.009713
Muscle tissue development	394	21	2.449627477	0.009713
Proteoglycan metabolic process	92	9	4.4960554	0.010243
Cell aggregation	25	5	9.191935484	0.010243

Regulation of cell adhesion	837	35	1.92185031	0.010668
Negative regulation of response to stimulus	1940	66	1.563576654	0.010668
Negative regulation of protein targeting to mitochondrion	6	3	22.97983871	0.010815
Regulation of cation transmembrane transport	399	21	2.41893039	0.010815
Cell adhesion mediated by integrin	74	8	4.968613775	0.011004
Plasma membrane bounded cell projection organization	1649	58	1.616532014	0.011004
Cellular response to endogenous stimulus	1505	54	1.649051549	0.011226
Cell projection organization	1690	59	1.604509448	0.011519
Striated muscle tissue development	376	20	2.444663693	0.01256
Metencephalon development	117	10	3.928177557	0.01256
Response to growth factor	750	32	1.960946237	0.012831
Regulation of transmembrane transport	622	28	2.068924385	0.013116
Anterior/posterior pattern specification	214	14	3.006707869	0.013346
Cell part morphogenesis	721	31	1.976074896	0.013617
Regionalization	352	19	2.480778043	0.013837
Steroid metabolic process	352	19	2.480778043	0.013837
Modulation of chemical synaptic transmission	470	23	2.249090597	0.013837
Regulation of trans-synaptic signaling	471	23	2.244315458	0.014085
Ossification	416	21	2.32007987	0.016364
Phospholipid metabolic process	479	23	2.20683211	0.017528
Cell projection morphogenesis	704	30	1.958508981	0.018471
Organic hydroxy compound metabolic process	607	27	2.04433491	0.018471
Angiogenesis	544	25	2.112117528	0.018525
Visual learning	45	6	6.127956989	0.018586
Glycerolipid metabolic process	484	23	2.184034258	0.019319
Positive regulation of transport	1013	39	1.769424896	0.019319
Response to lipoprotein particle	30	5	7.659946237	0.019319
Behavior	611	27	2.030951375	0.019413
Cell morphogenesis	1085	41	1.736725138	0.019607
Cytoskeleton organization	1553	54	1.598082795	0.019607
Chondrocyte development	31	5	7.412851197	0.021666
CGMP-mediated signaling	31	5	7.412851197	0.021666
G protein-coupled receptor signaling pathway, coupled to cyclic nucleotide second messenger	65	7	4.949503722	0.021893
Low-density lipoprotein particle remodeling	18	4	10.21326165	0.022283
Alcohol metabolic process	403	20	2.280877291	0.02432
Cellular response to lipoprotein particle stimulus	32	5	7.181199597	0.024435
Cellular response to growth factor stimulus	722	30	1.909681887	0.024485
Cerebellum development	109	9	3.79483575	0.025718
Positive regulation of cell differentiation	897	35	1.79329845	0.027101
Visual behavior	50	6	5.51516129	0.029078
Circulatory system process	632	27	1.963467232	0.029199
Regulation of cell differentiation	1738	58	1.533752181	0.029819
Regulation of signaling receptor activity	211	13	2.831638893	0.029859
Plasma membrane bounded cell projection morphogenesis	700	29	1.904043779	0.029859
Positive regulation of cholesterol esterification	9	3	15.31989247	0.030065
Cardiac muscle cell development	70	7	4.595967742	0.031113

Positive regulation of cellular component organization	1192	43	1.657941383	0.032158
Pallium development	188	12	2.933596431	0.033225
Developmental growth	640	27	1.938923891	0.033225
Organophosphate catabolic process	163	11	3.101573323	0.033736
Cardiac muscle tissue development	215	13	2.778957239	0.033736
Protein phosphorylation	1678	56	1.533815218	0.034035
Negative regulation of G1/S transition of mitotic cell cycle	115	9	3.59684432	0.034035
Locomotory behavior	191	12	2.887519	0.036817
Regulation of exocytosis	219	13	2.728200029	0.037593
Signal release	518	23	2.040680658	0.037593
Sterol import	10	3	13.78790323	0.037593
Wound healing	583	25	1.970826647	0.037593
Cholesterol import	10	3	13.78790323	0.037593
Sensory organ morphogenesis	274	15	2.516040735	0.037593
Cellular response to oxidised low-density lipoprotein particle stimulus	10	3	13.78790323	0.037593
Negative regulation of establishment of protein localization to mitochondrion	10	3	13.78790323	0.037593
Negative regulation of cell cycle G1/S phase transition	118	9	3.505399125	0.038115
Response to wounding	718	29	1.856310091	0.038206
Muscle organ morphogenesis	74	7	4.347537053	0.038206
Positive regulation of cell projection organization	365	18	2.26650464	0.038792
Regulation of cellular component biogenesis	1032	38	1.692313703	0.038792
Pattern specification process	460	21	2.098159187	0.041031
Cellular carbohydrate metabolic process	308	16	2.38751571	0.041258
Cardiac cell development	76	7	4.233128183	0.043236
Pre-B cell allelic exclusion	3	2	30.63978495	0.044712
Lymphatic endothelial cell fate commitment	3	2	30.63978495	0.044712
Regulation of kainate selective glutamate receptor activity	3	2	30.63978495	0.044712
Intestinal epithelial cell differentiation	23	4	7.992987377	0.044908
Actin filament organization	467	21	2.066709263	0.04674
Visual perception	227	13	2.632052011	0.04674
Regulation of NMDA receptor activity	39	5	5.892266336	0.04674
Regulation of nervous system process	149	10	3.084542109	0.049896

Supplementary Table 9 Gene Ontology Biological Processes enrichment analysis of DEGs in sporadic versus controls. Enriched Cellular Components for upregulated genes with layout as Supplementary Table 6.

enrichment_GO_MF_sPD				
Pathway	Total	Hits	Enrichment	FDR
Extracellular matrix structural constituent	186	21	5.188995838	7.81E-07
Extracellular matrix structural constituent conferring tensile strength	45	8	8.170609319	0.002426
Structural molecule activity	784	36	2.110393351	0.005572
Lipid binding	811	37	2.096804025	0.005572
Calcium ion binding	770	35	2.089076246	0.007124
Phosphatidylinositol-5-phosphate binding	20	5	11.48991935	0.009065
Phosphotransferase activity, alcohol group as acceptor	742	33	2.04402878	0.012828
Kinase activity	848	35	1.896920648	0.029172
Cell adhesion molecule binding	577	26	2.070973333	0.044748
Integrin binding	152	11	3.326029287	0.04622
Lysophosphatidic acid binding	8	3	17.23487903	0.04622

Supplementary Table 10. Gene Ontology Molecular Function enrichment analysis of DEGs in sporadic versus controls. Enriched Cellular Components for upregulated genes with layout as Supplementary Table 6.

Code	Mutation	Gender	age
JS48753	GBA1 E326K	M	59
MB240649	GBA1 E326K	M	63
CK290347	GBA1 E326K (HomoZ)	M	65
Control1		M	71
Control2		M	43
Control3		F	66
sPD 1		M	51
sPD 2		M	55
sPD 3		M	46
sPD 4		M	65

Supplementary Table 11. A description of PD patients and controls that participated in the study.

Enriched_CC_sPD				
Pathway	Enrichment FDR	nGenes	Pathway Genes	Fold Enrichment
Neuron projection	5.29473030811923e-08	71	1444	2.259790233
External encapsulating structure	2.11690474787198e-07	39	601	2.982408352
Extracellular matrix	2.11690474787198e-07	39	600	2.987379032
Synapse	2.11690474787198e-07	68	1443	2.165806005
Collagen-containing extracellular matrix	4.86618297337138e-07	32	446	3.297555331
Receptor complex	1.95233736374745e-05	28	422	3.04945727
Plasma membrane region	2.52289546973582e-05	58	1323	2.014861142
Integral component of plasma membrane	3.21771471059385e-05	74	1881	1.808089383
Intrinsic component of plasma membrane	3.77688878259881e-05	76	1965	1.77757531
Synaptic membrane	4.94196055440749e-05	26	401	2.979929209
Postsynapse	5.49151847425893e-05	36	682	2.426024028
Somatodendritic compartment	9.17552470013141e-05	43	913	2.164585026
Postsynaptic membrane	0.000111842	21	297	3.249674161
Plasma membrane signaling receptor complex	0.000236805	16	194	3.790488859
Postsynaptic specialization membrane	0.000514373	12	121	4.557984537
Fibrillar collagen trimer	0.000544	5	16	14.36239919
AMPA glutamate receptor complex	0.000544	6	26	10.6060794
Banded collagen fibril	0.000544	5	16	14.36239919
Synaptic vesicle	0.000563779	16	213	3.452370135
Neuronal cell body	0.000563779	28	535	2.405366295
Axon	0.000641308	33	691	2.194890528
Postsynaptic specialization	0.000767229	22	376	2.689130062

Enriched_CC_GBA1				
Pathway	Enrichment FDR	nGenes	Pathway Genes	Fold Enrichment
External encapsulating structure	0.000778125	36	601	2.334161014
Extracellular matrix	0.000778125	36	600	2.338051282
Endoplasmic reticulum lumen	0.006597521	22	328	2.613675214
Basal plasma membrane	0.006597521	19	265	2.793897758
Internode region of axon	0.006597521	3	4	29.22564103
Receptor complex	0.006597521	26	422	2.400842549
Collagen trimer	0.006988459	11	105	4.082311762
Podosome	0.009318753	6	33	7.085003885
Basal part of cell	0.009318753	19	283	2.616194014
Plasma membrane signaling receptor complex	0.009318753	15	194	3.012952683
Glutamatergic synapse	0.009318753	22	358	2.394652151
Collagen-containing extracellular matrix	0.012199488	25	446	2.184278104
Postsynapse	0.018865127	33	682	1.885525227
Basolateral plasma membrane	0.020220106	16	240	2.597834758
Synapse	0.020220106	58	1443	1.56626212
Basement membrane	0.029145175	9	97	3.61554322
Intrinsic component of plasma membrane	0.029145175	73	1965	1.447648377
Neuron projection	0.029145175	57	1444	1.538191633
Integrin complex	0.037748097	5	32	6.088675214
Basement membrane collagen trimer	0.037748097	3	9	12.98917379
Plasma membrane region	0.043672287	52	1323	1.531603259
Integral component of plasma membrane	0.045396107	69	1881	1.429430608

Supplementary Table 12. The top 22 dysregulated pathways in DA neurons derived from PD patients with E326K-*GBA1* mutations compared to control DA neurons and from sPD patients compared to control neurons. The highlighted pathways are shared between these 2 PD groups.

DEGs_GBA1	
**Gene ID**	**Fold change**
FZD10-AS1	-6.219631314
TMEM132D	-4.859769902
FZD10	-4.783255274
A2M	-3.148072168
LINC02228	-3.015944299
ZFP42	-2.840491192
LINC00906	-2.78224874
PTH2R	-2.66523942
STYXL2	-2.651384589
TLX1	-2.607025957
IGF2-AS	-2.595644756
IGF2	-2.592744497
XAF1	-2.55996299
PTPRC	-2.540601438
A2M-AS1	-2.535904127
FGF10-AS1	-2.509438847
ASNSP1	-2.498889595
PRAL	-2.483678851
NECTIN3-AS1	-2.478304297
HRH1	-2.462200449
DGAT2L6	-2.368540165
LINC02211	-2.323216726
HLA-F	-2.275000152
FOXD2	-2.244346811
NT5E	-2.235040009
KLHL41	-2.204416616
FGF10	-2.149258651
SGCG	-2.14685133
RPL23P8	-2.13322205
IL6	-2.099670388
PTPRT-DT	-2.086833539
VCAM1	-2.016045756
GDA	-2.013158691
THORLNC	-1.970951235
CTHRC1	-1.965397986
PTPRT	-1.953346799
STRIT1	-1.951272205
DAPK2	-1.946777879
UNC5B	-1.945272034
ADAM32	-1.940112185
SEMA3D	-1.930448139
INSRR	-1.912759731
C4A	-1.907964261
PTPRE	-1.906169695
F2RL1	-1.889008801
B3GNT7	-1.866717125

LRRK1	-1.866392781
RPL10P6	-1.863297619
COX20P1	-1.859845782
SFRP4	-1.84819034
CDCP1	-1.845022405
TMEM158	-1.842429288
TNFRSF9	-1.824689378
MMP1	-1.812595383
COL15A1	-1.808055739
RBM46	-1.807043227
PTGDS	-1.785274479
WNT2	-1.783946321
IL20RA	-1.772177062
DES	-1.770689366
TBX15	-1.762615346
SCRG1	-1.756716631
LAMC3	-1.752563202
RNASE1	-1.752115388
LGALS3	-1.744625635
AGAP7P	-1.739069066
AGT	-1.71487689
HPR	-1.711794996
RUNX1	-1.710864359
SOCS3	-1.706667366
HS3ST3A1	-1.705258479
IFI16	-1.700997257
ARHGEF35	-1.694218364
MMP13	-1.692793713
FBXO32	-1.692586413
HGF	-1.690364272
A2MP1	-1.690357751
TBX18	-1.68746758
TNN	-1.681277771
GDF15	-1.673786631
CAPN6	-1.659344504
YPEL3-DT	-1.657204238
TRIB3	-1.652090576
SBSPON	-1.644278256
TBC1D3E	-1.644088511
MAP1LC3C	-1.644012575
A4GALT	-1.64338875
PCK1	-1.634363994
TMPRSS2	-1.62551905
MTND4P12	-1.623316346
ALPK3	-1.620217827
DIRAS3	-1.607924332
SDR42E1	-1.60586017
LINC02485	-1.604715608

CAV2	-1.600047883
SPHK1	-1.593519206
ALDH1A3	-1.588467969
LINC01139	-1.588065053
F11	-1.572862499
SAT1	-1.565064385
ADA2	-1.563332311
KLF2	-1.558730704
JUNB	-1.550562881
FKBP5	-1.548827984
ALDH1A3-AS1	-1.546500117
SUCNR1	-1.544198872
C4B	-1.541712006
FOXQ1	-1.538332267
SYCP1	-1.529669732
HS3ST3B1	-1.526628855
MCOLN2	-1.526109149
GLIPR1L2	-1.523679277
ARHGEF35-AS1	-1.516681315
FOXC1	-1.514632504
PAPLN	-1.509098203
FOXD2-AS1	-1.508280354
KIAA1755	-1.504995339
CDH5	-1.501440324
OSMR	-1.49122776
NPFFR2	-1.484316397
CD34	-1.483637984
VSX2	-1.482646024
SSXP10	-1.482480342
LSP1	-1.481698407
RGPD2	-1.481391556
CAV1	-1.481013048
PRR15	-1.471211272
INSYN2A	-1.469845511
ITGA7	-1.466312435
NCBP2L	-1.461659463
LINC00390	-1.4607363
RAP1AP	-1.459806336
ADGRA3P1	-1.449499606
IL4R	-1.44843203
MYH14	-1.444790275
RUBCNL	-1.437618479
ZNF623	-1.429094737
LCNL1	-1.42534735
RUNX2	-1.418852375
ARHGAP36	-1.418491759
AFAP1L1	-1.413198147
PTP4A3	-1.405668344

HAP1	-1.405575021
SLC29A1	-1.404184645
BHLHE40-AS1	-1.3901367
STEAP1	-1.383345606
F7	-1.382258894
BHLHE40	-1.38058185
PHLDA1	-1.375706193
ARSJ	-1.372546863
TSPEAR-AS1	-1.371769586
CLDN1	-1.363833284
FILIP1L	-1.363101431
HCN1	-1.358096738
GADD45B	-1.356981014
MX1	-1.354048359
ANKRD18CP	-1.353090292
MEF2C	-1.341329208
DOCK10	-1.334838356
SUNO1	-1.332963249
MYLK	-1.332594184
ELMO1	-1.332498276
CNMD	-1.3297641
GLI1	-1.323429761
NIPAL2	-1.317779028
ZNF558	-1.307281817
DCN	-1.306938862
TSPEAR-AS2	-1.304842265
RNF125	-1.304512258
SYBU-AS1	-1.301551011
LIF	-1.297989956
EPCAM	-1.295677519
MIR503HG	-1.292583657
KCNH5	-1.280794524
ITGB4	-1.270215638
ARHGAP10	-1.268827324
RGS6	-1.26485957
P4HA2	-1.256818291
SLC22A8	-1.245774459
#N/A	-1.245374393
CDKN2A	-1.245255729
AKR1E2	-1.242105046
PTX3	-1.236251695
CRACDL	-1.231009465
PHYHD1	-1.229045026
WWTR1	-1.205260662
ZNF365	-1.200267356
KANSL1-AS1	-1.195789579
DIAPH2	-1.187340199
MYO16	-1.185501917

CMTM8	-1.185392341
IRAK3	-1.178317336
GIPC2	-1.174884083
ZCCHC24	-1.167123289
NABP1	-1.160952104
LINC01852	-1.155051196
CA5B	-1.152758138
PHLDA1-AS1	-1.144896158
SLC12A2	-1.143558633
INE2	-1.142855478
CPA4	-1.141814094
MBP	-1.141713588
SMOX	-1.138407613
ITGB3	-1.136664929
C1S	-1.124578595
AHR	-1.123362174
HEPH	-1.1208498
NRP1	-1.119569055
RGS1	-1.115957252
KLF13	-1.108707207
EDIL3-DT	-1.107883931
GBP5	-1.107276964
WASH7P	-1.0970009
KCTD12	-1.090862237
ITGA3	-1.086877437
BEND6	-1.086314585
RHOJ	-1.080671551
UQCRFS1P1	-1.076076892
AKAP12	-1.072856516
NDNF	-1.065976981
CRPPA	-1.06483158
BCL6	-1.063739425
PNMA3	-1.063731056
RIOX2	-1.059138423
TRMT9B	-1.059042181
ULK4	-1.058713829
UBE2V1	-1.052328214
IL6ST	-1.051598581
NPIPB3	-1.041717545
METTL7B	-1.034215947
MYO1D	-1.033464857
ELK3	-1.029167254
ARID5A	-1.028020722
WWTR1-AS1	-1.015732838
TAFA5	-1.014822477
PRKD1	-1.005228285
CDKN2B-AS1	-1.003298269
MET	-0.992317436

PWRN1	-0.989573401
CADPS	-0.972389214
GOLGA6L10	-0.969845431
MAN1C1	-0.969157358
ENDOD1	-0.965508721
FUT4	-0.965336719
SH3PXD2A	-0.957431211
PDZD2	-0.957232722
SBF2	-0.957212719
CIART	-0.950378808
MARCHF1	-0.948706553
LMCD1-AS1	-0.944829704
USP25	-0.941413705
SVIL	-0.936467936
PDE7B	-0.929100564
ABCA1	-0.92806584
SNTA1	-0.926059963
ZFX	-0.925568866
PRRG1	-0.919114572
PCSK1N	-0.914563291
COPZ2	-0.913409449
TSC22D3	-0.909331524
PDE3B	-0.905070059
PAPSS2	-0.903941341
COL4A2-AS2	-0.896636964
ELL2	-0.889623407
CUBN	-0.884623222
EIF5A2	-0.877912936
COL4A1	-0.875603209
PROX1	-0.875393585
ARID5B	-0.875177197
FGF2	-0.87499225
DCAF4	-0.874583571
UBE2Q2P2	-0.864471475
MAGI2-AS3	-0.860330296
SPIRE1	-0.849449494
ST20	-0.849312811
EPM2A	-0.847907662
LONRF1	-0.845748952
CAMK1D	-0.840220431
KICS2	-0.839449092
TFRC	-0.838389045
RDH10	-0.828702561
PIP4K2A	-0.826577747
PELI1	-0.814307993
SBF2-AS1	-0.813391689
RASSF8	-0.809302257
MID2	-0.807799199

PRELID2	-0.803793192
STK3	-0.782924056
TMEM200C	-0.781674674
CHORDC1	-0.777497423
UTP15	-0.775809262
SLC4A7	-0.772842362
RETREG1	-0.772751272
SUSD1	-0.770097154
CASC15	-0.769311453
EEA1	-0.768026415
NEDD9	-0.767215458
SPART-AS1	-0.765407382
HSPA9	-0.764129451
SUGT1P1	-0.756940549
DGKH	-0.749693261
SH3BP2	-0.748422573
IL13RA1	-0.742253453
CPNE3	-0.741621698
UACA	-0.741047383
COL4A2	-0.740340511
DDX60	-0.739464133
SH3RF1	-0.732531371
ASPH	-0.732079301
IRS2	-0.727672295
EFR3A	-0.727245338
KLHDC1	-0.725722606
MLIP	-0.723909397
MPZL3	-0.722846812
TCF7L2	-0.719826193
ALG13	-0.719715423
SNX9	-0.713448177
HYCC1	-0.71193644
STAT1	-0.707120901
SPRED1	-0.703373541
EOGT	-0.690660275
IRF1	-0.69031641
ZEB2	-0.687045455
JUND	-0.686357486
VMP1	-0.686103937
PPP2R5A	-0.684248294
TMEM65	-0.672972361
TXLNG	-0.669471006
SLFN5	-0.667699453
B3GNT9	-0.665659096
SNX14	-0.657246013
GPR155	-0.654800804
GNA13	-0.65362053
GOLGA2P7	-0.653136518

DNAH9	-0.650283197
CD164	-0.647444443
UGGT2	-0.645523949
STAT3	-0.643219805
ZNF621	-0.630360015
CCSER2	-0.630309931
SIRT1	-0.627661264
HACD2	-0.627569843
SLC25A37	-0.624416995
FNIP1	-0.623166609
LRRC58	-0.622140035
OSGIN2	-0.620970157
GARIN5A	-0.619628891
C12orf75	-0.614652142
CDV3	-0.614552357
CDC42EP3	-0.612418743
RNF217	-0.608172703
ZEB2-AS1	-0.607331073
CEP85L	-0.603203667
EMC10	-0.597707945
KIF13A	-0.597589225
EVI5	-0.59676024
ZNF419	-0.596248581
CTDSP1	-0.595210934
FYCO1	-0.59365428
ACSBG1	-0.589299169
AP1AR	-0.5851073
YES1	-0.584428721
LIMS1	-0.575404833
ERO1A	-0.57210838
GLRX2	-0.571934432
TLK1	-0.571763893
MTMR10	-0.569904245
PTPN12	-0.56810106
PRMT3	-0.564248116
GBE1	-0.563800712
IDH3A	-0.560843909
PURA	-0.559156899
GALC	-0.558715468
SLC19A2	-0.555487272
SHLD2	-0.55432177
CAPRIN2	-0.553410387
KDSR	-0.552856289
FAM20C	-0.552538371
FAM91A1	-0.533679258
ARF6	-0.532135161
EIF5	-0.531782075
ALG10B	-0.528068399

GCFC2	-0.525943496
PAK2	-0.525839924
TCAIM	-0.524122731
OSGEP	-0.524044007
RCL1	-0.520608102
PPP1R2	-0.520577809
CYP2U1	-0.519584519
CHMP2B	-0.518671636
PARD3	-0.511356329
DENND1B	-0.511021508
DDX21	-0.510620464
RAP2B	-0.503551471
USP45	-0.503286439
RNF149	-0.501267425
ERBIN	-0.500299746
PPRC1	-0.499999214
ARFGEF2	-0.496267488
MTFMT	-0.494604215
ADO	-0.494322115
CDK13	-0.491201694
ANKRD28	-0.487985383
PPP1CB	-0.487753834
MAP4K5	-0.485949553
BEND7	-0.484525665
GOLGA5	-0.482652886
ANKRA2	-0.482015493
ABHD5	-0.477724188
TMEM33	-0.477589683
PLEKHG1	-0.475764271
PNPT1	-0.474490469
MBTPS2	-0.474004001
ZFAND5	-0.472191999
TARS3	-0.467319326
FYTTD1	-0.466403634
WASHC4	-0.465326541
TDRP	-0.463422018
ATP7B	-0.455621562
BMP2K	-0.454828745
EIF2AK2	-0.444433725
IQCB1	-0.441952483
AGA	-0.441063721
ZDHHC2	-0.438763838
PDCD6P1	-0.433336651
TJP1	-0.431062902
PTAR1	-0.431009816
ZMYND11	-0.430026329
VRK2	-0.428601307
JAK1	-0.421571892

ATP13A3	-0.419956227
GNG7	-0.418508563
ME2	-0.416676595
NOC3L	-0.416219337
BTBD1	-0.414977219
USP6NL	-0.414214831
WNK1	-0.407447833
SOCS6	-0.403896638
TMEM181	-0.399123606
NCOA3	-0.3975133
PJA2	-0.397309647
ZNF275	-0.397213393
AFF4	-0.394803043
ARL13B	-0.390445008
HECTD1	-0.388683121
DAZAP1	-0.386153194
SENP6	-0.385285592
SERAC1	-0.380654584
TDRD3	-0.3783159
SH3YL1	-0.36481801
TMEM192	-0.363827802
DNAJB14	-0.358767315
DENND4C	-0.347368119
SRSF6	-0.347097487
SUSD6	-0.339637152
UBE2E3	-0.339515362
ARL10	-0.328576812
RIC1	-0.325761403
DNAJC13	-0.315993467
MTDH	-0.314205185
ACBD5	-0.313864033
EVC	-0.294416901
PGM3	-0.285045979
CMTR2	-0.221082353
PSMC5	0.303087424
MED24	0.326025256
SGTA	0.365730608
DNASE1	0.367934929
AP2M1	0.37104587
B3GNT4	0.372672227
AP1B1	0.398521604
FAM222B	0.405428179
RBM10	0.406900727
PPP1R7	0.413907185
POLR2F	0.416273396
FNTB	0.428986652
PLGRKT	0.432351741
WRAP53	0.437488003

PRMT7	0.448412776
VDAC3	0.44851658
MOSPD3	0.449321736
AP1M1	0.450464568
PAFAH1B3	0.453625932
C12orf57	0.467765098
TUT1	0.477701682
MRPS11	0.477869326
HSD17B10	0.479747858
CSE1L	0.486505608
ELOB	0.48694552
RAB30-DT	0.490388278
SUN2	0.499596777
ARRB2	0.502028646
ZNF607	0.506376835
POP5	0.509643612
RETSAT	0.511384991
SDF2	0.522727067
LMNB2	0.52514828
NUDT2	0.53294289
LSM3	0.534727091
PCSK7	0.535716133
PLCD4	0.538416616
NDUFS5	0.538515141
B3GNTL1	0.539591256
PTGR3	0.541951009
NCAPH2	0.543543204
CDK5	0.549008964
CPT1C	0.551610363
PAPSS1	0.55351744
DOC2A	0.559523587
ATP5MC1	0.56159251
C1orf35	0.563774798
NAT14	0.567280805
CCDC28B	0.584376614
MCF2L	0.594088232
SUFU	0.597121076
COG4	0.603175292
ZNF107	0.60459073
WNK2	0.6049389
HIRIP3	0.614523608
CSRNP1	0.618639857
BICDL1	0.63266218
MZT2A	0.632853177
CKB	0.635356749
UBL5	0.640743336
TOR2A	0.641043405
GSTCD	0.644724906

CXXC4-AS1	0.645259548
TFPT	0.65509352
ACYP1	0.66095251
ADAMTS9	0.661734806
CXXC4	0.67202628
IP6K2	0.674660081
PASK	0.678378829
DDB2	0.680817564
TMEM107	0.68176776
IGDCC3	0.68881525
ECH1	0.69290959
CDH24	0.693512088
CEP78	0.694491747
LSM4	0.695251689
DYNC2I2	0.696313797
H3-3A	0.704047671
ETHE1	0.712748417
MCPH1-DT	0.712854328
GPR19	0.716313914
KANK3	0.736015843
PLCH1	0.739959877
DPYSL5	0.751777079
CHST6	0.762072521
FRG1HP	0.776439875
TSHZ1	0.779634605
KLHDC8A	0.786407706
MPP2	0.792847734
GLB1L2	0.801938551
CELSR3	0.802721316
FRRS1	0.806933271
KLHL3	0.810848747
FBXO16	0.814483519
NUDT1	0.816132635
C1QTNF3	0.823680717
LINC00205	0.824224834
TSPAN10	0.825270755
YJEFN3	0.836050884
CHGB	0.839294555
ATAD5	0.839309665
PCDHGC4	0.843410538
CHKB-DT	0.843470119
FAM81A	0.85073086
UBL7-DT	0.850877453
RRM1	0.857741142
SPIRE2	0.860338417
SSBP3-AS1	0.867280699
SLC5A9	0.871781979
LINC01719	0.876812863

PCAT1	0.87744432
PTGR1	0.878019958
KCNAB2	0.880888131
TMEM163	0.884850745
ARID3B	0.887229151
CASC9	0.890719795
CEP128	0.894809273
RRM1-AS1	0.894857695
PRIM1	0.915290635
FXYD6-AS1	0.926199062
SLC9A3-AS1	0.934243624
FXYD6	0.935096461
CASZ1	0.936261363
THAP7-AS1	0.952824377
WDR38	0.954201093
ID2	0.957096908
TMEM263-DT	0.959225299
DHRS11	0.960292259
GABRA5	0.962512317
SRCIN1	0.968731694
LINC02696	0.970556629
GPER1	0.984064934
PEX11G	0.998447314
FAM88B	0.998583126
SHD	1.003610372
UBAC2-AS1	1.005769217
SLC9A3	1.007615346
TLE4	1.008199822
ADAMTSL2	1.011562679
MYB	1.013000037
PTPRD-AS1	1.01331059
ST8SIA5	1.021298306
SLC25A53	1.021647964
BAHCC1	1.026365336
ALMS1P1	1.03015414
TPPP	1.030641092
COL5A3	1.032804311
CORO1A	1.035576426
OXA1L-DT	1.039044282
HSD17B8	1.04379869
RENO1	1.045647236
FGFR2	1.053011249
CHST8	1.065894604
CHRNA5	1.069361874
ZFHX4-AS1	1.071616332
PDCD6-DT	1.071670804
MOB3B	1.072466021
ETFB	1.075425811

OCA2	1.086399444
SLC16A9	1.08968305
ZNF503-AS2	1.095342742
KIAA2012	1.097073743
CCDC74B	1.099489334
TYW1B	1.099962964
PYGM	1.100957967
SLC12A8	1.109118466
BRCA2	1.118300883
MTFR2	1.12118159
TMEM139-AS1	1.124525874
METTL7A	1.126541404
RAB3C	1.128587282
LRRN2	1.130762787
ZNF474	1.137041616
RPRM	1.138799967
TBC1D22A-DT	1.142105012
ADAM8	1.142582485
DNASE1L2	1.143099749
TEX15	1.150856495
S100B	1.151087538
GALNTL6	1.151584905
NMB	1.154244245
RMI2	1.155354829
DNM1P51	1.159450241
ANKFN1	1.172795959
MIDEAS-AS1	1.176782443
FOXP1-DT	1.177054137
ZNF503	1.178289496
FXYD7	1.181298023
LRRC37A4P	1.188521467
TMSB15A	1.188783814
PCSK2	1.210396105
SRL	1.21075521
PCSK1	1.216305464
HGFAC	1.225858292
RAPGEF5	1.229789977
EPHA7	1.234505587
IFI27	1.24518134
ZNF367	1.248686259
ID1	1.258882772
ELFN2	1.259493435
LINC03013	1.261273861
CHRNG	1.261819498
AMZ1	1.264628057
ADCYAP1R1	1.268261492
#N/A	1.270972363
DLL1	1.286477104

DLL3	1.287024144
GALNT12	1.290531219
PKD2L2-DT	1.291423219
TAT-AS1	1.295681095
HAS1	1.304106525
PARPBP	1.310049291
SLC1A1	1.31919809
GDF5	1.322377235
ST6GAL2	1.32902376
RAB3B	1.344334032
LRRC63	1.353392105
BRINP2	1.353829822
IRX2	1.354636684
DDIT4L	1.362191761
RPE65	1.371652959
REM2	1.37188075
GDF5-AS1	1.372753974
TSPAN15	1.374141209
LARGE2	1.374538024
FHAD1	1.375544429
A4GNT	1.378264805
LINC01517	1.379027975
LRRC74B	1.380232289
SUSD2	1.382062928
SMPDL3B	1.382635226
FUT9	1.388799282
ZNF528	1.394841792
WFIKKN1	1.400578633
LINC02593	1.40302091
TFCP2L1	1.412423197
DKK2	1.419268393
SLC28A2	1.419441302
LRRC52	1.421541475
BMP4	1.424337916
PTGER2	1.425085334
MMRN2	1.427487313
PAH	1.429378328
PRDM16	1.430901872
COL6A6	1.436645098
WSCD1	1.441806749
NOTUM	1.441853934
MGAT4C	1.450530503
GAS1	1.451652076
CCBE1	1.458042768
CACNA1S	1.461496943
C22orf15	1.468136875
RSPO3	1.477121583
DACH1	1.478050143

SEMA3F	1.479627194
LEMD1-DT	1.479762302
RDM1	1.487604821
GMNC	1.496043394
ITGB7	1.497122465
FREM3	1.498269095
TM4SF18	1.505013699
ZNF560	1.507431847
EIF2S3B	1.508254341
MYO18B	1.511219796
PCDHGA3	1.516669977
RPL24P8	1.517467401
GPSM3	1.517936023
LINC00664	1.521258268
AKAP3	1.524230516
LERFS	1.527932826
ZIC4	1.528043588
LINC02958	1.530369804
NRGN	1.535687012
RNF5P1	1.536824571
ZNF887P	1.541951522
RD3	1.545007798
NOSTRIN	1.545995421
ASCL5	1.546157942
UFL1-AS1	1.548372226
BMP5	1.552259478
LMX1A-AS1	1.556973928
TRABD2A	1.557181029
ADAMTS17	1.557913675
CHRND	1.559994773
KLF2P4	1.562049767
CASQ2	1.564560276
CXCL14	1.564669858
BTBD17	1.564973159
NRAP	1.573957394
OTX2	1.581398786
MAP3K19	1.581518153
PRRT1B	1.582587784
HSD11B2	1.587185989
GALNT14	1.588128918
LRRIQ3	1.590313615
CARTPT	1.590764296
NFE2	1.591663963
TNFRSF11B	1.592940875
KLK6	1.594610785
CA4	1.596179795
GAS1RR	1.59884307
URB1-AS1	1.59967141

FAM181B	1.601447674
SLC28A2-AS1	1.602480152
ECEL1	1.603084784
ZNF528-AS1	1.603868683
LMO3	1.607087776
ASCL1	1.608175811
KIF19	1.608425964
CMAHP	1.616048256
AOC3	1.618241311
LINC02931	1.620935196
VIT	1.628970801
H4C5	1.629543097
UBE2T	1.636043157
CAPN13	1.640335285
COL9A1	1.64150153
TNFSF10	1.648689173
VGLL2	1.651879099
FOXN4	1.661039334
FOLR1	1.661816808
CLIC6	1.668745241
LINC00881	1.67479678
GRIN2C	1.681736448
ANKRD20A5P	1.690906178
CKM	1.69207464
CAPSL	1.697047666
BARHL1	1.697310254
POU5F1P6	1.704836547
TNNC1	1.710380329
NCKAP1L	1.721266594
LINC02381	1.726941557
GABRR1	1.732660927
FAR2P4	1.751547005
MKRN3	1.760871388
MALRD1	1.764512849
MLLT10P1	1.766717266
ZNF454	1.786516418
CEACAM16-AS1	1.798231704
NR4A2	1.816931468
MFNG	1.850331682
RSPO2	1.852188983
GRM6	1.879190326
REM1	1.894570801
DMRTA2	1.916509823
PRDM16-DT	1.92337794
RSPO1	1.926229116
TNNT1	1.952161507
NTS	1.957902436

FMO1	1.965635309
LINC02899	1.966761581
WNT10B	1.988431292
TEKT4	1.989213035
LMX1A-AS2	2.015859329
HERC2P3	2.049834175
PTGS1	2.053927796
ERMN	2.05520577
HPD	2.077250236
LIX1	2.086735672
PMCH	2.127174261
FAM66A	2.137090667
NMU	2.214302231
LMX1A	2.247914893
ZNF229	2.360352227
MED15P8	2.471569856
GDF7	2.498344739
CLDN19	2.545726835
TMEM72	2.580516485
NEK2P4	2.6262238
PCDHGA7	2.690927409
PLD5P1	2.939165402
MSX1	2.94429728
PNMA6B	3.130084961
WNT3A	3.214904821
NF1P8	3.413212737
HSD17B7P2	3.674384193

**DEGs_sPD**	
**Gene ID**	**Fold change**
CHL1	-10.09932654
DPP6	-7.261566405
SGCA	-5.550136322
AL591846.1	-4.883993967
AL139158.2	-4.493436103
RPS2P5	-4.485354859
HLA-C	-4.481203518
CHMP1B2P	-4.437203192
LIF-AS1	-4.388587347
AGAP7P	-4.356916316
RPL13P12	-4.240342285
FEV	-4.110690711
AC011352.3	-3.818066064
AC087457.1	-3.781309081
MTND4P12	-3.747285337
SCGB3A2	-3.629844516
TRIM22	-3.6283473

AQP9	-3.589468285
EN1	-3.487699255
TEX26-AS1	-3.290146879
AC044860.1	-3.201089395
NPIPB15	-3.1890386
SCN4B	-3.187570056
AC011483.2	-3.171076748
IRX6	-3.15015361
LINC01158	-3.126499279
U2AF1L5	-3.117903895
AL132709.1	-3.090198923
AC254629.1	-3.025732698
GPR132	-3.016557361
AC105105.1	-3.00834832
IGFL2-AS1	-2.999091603
HGFAC	-2.91085576
AC002454.1	-2.867492777
MIR210HG	-2.816437961
AC055716.2	-2.790180559
FUT9	-2.758761536
INPP5D	-2.74683772
LRRC37A6P	-2.667975808
AC005180.2	-2.64143726
CBSL	-2.605556228
NPTX1	-2.604128731
AL391117.1	-2.570620216
AC011511.5	-2.548639188
DIO3OS	-2.540273733
PRR27	-2.516101713
ANKRD1	-2.496766206
VGF	-2.49641044
CCL2	-2.473125088
MUC3A	-2.469596047
IRX1	-2.460377642
OTX1	-2.433498088
RSPH10B2	-2.426600026
NOMO3	-2.399264857
LINC00672	-2.383663131
AC016595.1	-2.378425677
RGS5	-2.359900711
PAPPA-AS1	-2.355914061
FST	-2.352234936
BCAN	-2.320095002
AC009139.1	-2.315749057
AL355990.1	-2.310485601
MTTP	-2.300893775
POU3F3	-2.296037178
PKD1P5	-2.232087638

TNFRSF10A-AS1	-2.222390562
LINC01943	-2.175511522
SMTNL1	-2.154547978
C16orf89	-2.139588116
ANO4	-2.130075845
KCNMB2-AS1	-2.100330018
NETO1	-2.092741564
PVT1	-2.075536426
SGCD	-2.074995524
SLC6A17	-2.07352238
CALCR	-2.064774916
PXDNL	-2.06050137
PI15	-2.051249719
AC004264.1	-2.050869626
AC015909.1	-2.048259648
ELMOD1	-2.045665522
PDE1A	-2.041611256
CSPG4P12	-2.03480753
HMGCS1	-2.03257476
AC144831.1	-2.029387224
CXCL12	-2.024493049
IRX5	-2.02045371
LIF	-1.984773373
PTPRN2	-1.983603017
BEGAIN	-1.980891964
PLA2G4A	-1.978824089
SH2D5	-1.963571865
LINC01759	-1.961698635
COL1A1	-1.945449647
MICA	-1.933516953
HAP1	-1.93097035
CDKN1A	-1.927886095
IL11	-1.907252783
CSGALNACT1	-1.866990921
MSMO1	-1.857222162
PLPPR5	-1.855528627
PLA2G3	-1.843285835
AC104794.2	-1.824798326
FGFBP3	-1.805598417
COL5A3	-1.8023133
AL391988.1	-1.800162151
PIK3R3	-1.798376602
AL359198.1	-1.784816423
GRID1	-1.782390751
SEL1L3	-1.780169071
AC025180.1	-1.776504519
RGS20	-1.772849141

PRPH	-1.769439674
OPRD1	-1.768507855
AC125611.4	-1.761398721
IFNLR1	-1.757173302
PRSS12	-1.72949717
COL3A1	-1.721823726
MC1R	-1.71631781
CRABP1	-1.711591659
NES	-1.706562367
GNG4	-1.70457342
AC011270.2	-1.695426668
CACNG8	-1.685301037
RNASEH1-AS1	-1.679836698
FABP7	-1.665198131
SHISA9	-1.662730166
PHLDA3	-1.662658755
ASTN1	-1.65812269
DHCR24	-1.63496835
LINC01963	-1.62966121
GPR27	-1.629171943
AC114803.1	-1.62343718
CDH11	-1.621058031
FDXR	-1.612982999
MMP17	-1.603945554
THY1	-1.596620492
TGFBI	-1.589581439
FSIP1	-1.58753774
RAB33A	-1.58499044
RPSAP44	-1.580308988
HPCAL4	-1.578319491
C1S	-1.575633754
AC020934.1	-1.574858349
FNDC10	-1.57198275
DPPA4	-1.571981673
COL14A1	-1.566912272
AC124798.1	-1.564258496
BMPR1B	-1.56321218
IGSF9B	-1.550411317
LRRC10B	-1.53435737
OPN5	-1.534012792
PDPN	-1.52429262
ALPK2	-1.512987946
AC037198.2	-1.510716537
CILP2	-1.494585
FBXO41	-1.488949333
OGDHL	-1.486513334
IGFBP3	-1.485921773
AL139384.2	-1.484240365

IDI1	-1.483853772
PIM2	-1.479586075
LOXL1	-1.478342301
VWCE	-1.478266764
CLEC18B	-1.476892742
COL11A1	-1.471653473
LOXL1-AS1	-1.469578509
LIPG	-1.468973539
KCTD1	-1.460421984
NSDHL	-1.459574747
EPHA2	-1.45451739
SLC2A6	-1.450840974
PRSS35	-1.441167471
ELOVL6	-1.440958965
VWA5B2	-1.43920959
PPP2R2C	-1.436717511
CYGB	-1.434455183
HMGCR	-1.428781748
RAB3B	-1.424346386
STOM	-1.41388375
LGI2	-1.411984232
NEFL	-1.399978283
PNPLA3	-1.396307066
AC245297.3	-1.3816229
AL513217.1	-1.368934383
APOE	-1.361654203
B4GALNT1	-1.3611952
AP003396.5	-1.344265663
TFPI	-1.340299949
SCRG1	-1.333361258
CADM2	-1.32237877
AL049629.1	-1.319494595
SFRP2	-1.318728333
SYT5	-1.314806986
DHCR7	-1.306839238
LPAR1	-1.304375862
PEG10	-1.301918514
GNG11	-1.296957963
KCNK9	-1.296483137
KLHDC9	-1.295582628
AP000662.1	-1.292912293
CHRNA5	-1.288618188
AC007362.1	-1.280824897
P2RX6	-1.274983507
GRIA4	-1.266648509
EPN3	-1.264573572
FDFT1	-1.256762581
OLFM2	-1.252720512

DNM3	-1.252105967
METRNL	-1.248184263
UPP1	-1.243272055
AC008734.1	-1.233407624
CCDC184	-1.227353212
GAP43	-1.226262779
PRKX	-1.226260733
ASB16-AS1	-1.2257646
CRYAB	-1.220455784
SETD9	-1.216712213
Z99774.1	-1.205761559
MIR9-3HG	-1.205659516
NCAN	-1.203797259
SQLE	-1.199714323
SOX1	-1.188725352
STARD4	-1.187737707
RFLNB	-1.184557914
CRTAC1	-1.183784636
ITGB5	-1.17694642
CDK6	-1.173847269
NCR3LG1	-1.166390044
SC5D	-1.156916882
AL592078.1	-1.154541574
PLK2	-1.153934113
SERINC2	-1.149626657
AC104365.3	-1.14961789
EHD4	-1.143925332
LHFPL6	-1.143265446
TTC9B	-1.136757261
CDK5R2	-1.119563286
AC020659.1	-1.114818948
CERKL	-1.1123503
SYNDIG1	-1.111238361
PCA3	-1.109896138
GAL3ST4	-1.103811417
SLC26A10	-1.102736986
AC092118.2	-1.100474394
POU3F2	-1.081639757
NACC2	-1.079866458
FTH1	-1.078250413
BAG3	-1.065071073
SCD	-1.064193563
MDGA1	-1.060166007
TFPI2	-1.057420752
SLC26A6	-1.05589161
ABLIM2	-1.055037114
TSPAN11	-1.052012974
KCNQ2	-1.048006592

FNDC4	-1.042096902
PRRG1	-1.040748373
AC080038.1	-1.040402306
AL929472.3	-1.03008628
LINGO1	-1.028351207
VEPH1	-1.026801257
ZNF558	-1.021376894
GNGT1	-1.014487056
CYFIP2	-1.012945468
PNP	-1.009905801
EPB41L4B	-1.00549355
PCNA	-0.993136934
AC006504.5	-0.991318589
PANK1	-0.989626102
FSTL3	-0.987135924
PRUNE2	-0.98653575
Z97832.2	-0.981709733
RNF175	-0.97090457
AC068987.4	-0.968043506
TMBIM4	-0.966184729
NALCN-AS1	-0.961493375
CALD1	-0.957989321
NETO2	-0.955097565
AL158151.1	-0.947981453
C15orf59-AS1	-0.945188271
TMEM74B	-0.940442875
NMNAT2	-0.939853918
PRCP	-0.938688324
ACSS2	-0.937395584
COTL1	-0.936803295
CSTF3	-0.918636407
TMEM97	-0.917343232
PAK1	-0.915611954
AC113383.1	-0.909745723
AL645608.1	-0.909000468
DIABLO	-0.908648403
HCFC1R1	-0.907252338
PLXNA4	-0.901302801
TENM4	-0.892011722
TANC1	-0.888609725
TP53I3	-0.881299544
RPS27L	-0.875110384
AC007319.1	-0.875095741
BDNF-AS	-0.853268844
UBE2N	-0.848956049
AC005726.5	-0.838909221
CACNG7	-0.837746841
PODXL2	-0.83627828

SLC6A15	-0.832104645
SAP30L-AS1	-0.829343103
SMAP2	-0.829187701
WLS	-0.823688395
ELAVL3	-0.822551908
TIMP2	-0.816164391
EIF5A	-0.811898729
SERPINF1	-0.811372307
TREH	-0.811162049
AL121753.2	-0.796167277
NT5DC2	-0.795376991
RFTN1	-0.783653216
ACAT2	-0.780247977
AGAP2-AS1	-0.779587886
TDRKH	-0.779034307
RND2	-0.763467102
CHRM4	-0.758370947
BSCL2	-0.755107767
MMP24OS	-0.75069641
PODXL	-0.749486138
MFGE8	-0.745173274
SCAMP5	-0.744958566
GALNT17	-0.741075179
SH3BP4	-0.736902286
AC005786.3	-0.734650952
AC010127.1	-0.731600818
KCND3	-0.723325309
NECTIN2	-0.718918277
ATP6V0E2	-0.718153052
DBI	-0.711772107
IL17RB	-0.704686937
FOXH1	-0.704443814
AL391422.3	-0.682458435
GPR39	-0.67779584
IGSF8	-0.677782842
FCHSD1	-0.671731281
KLC3	-0.661533677
IGF1R	-0.655148729
ANKRD9	-0.645785695
TUBB2B	-0.64183188
BRD7	-0.636825385
TCTE1	-0.634897533
PMEPA1	-0.629847788
CAPNS1	-0.623529559
RELL2	-0.619092356
TMEM129	-0.617658417
AC024896.1	-0.612953274
LRRC8C-DT	-0.590349008

AL451085.2	-0.582336307
ATP6V0E2-AS1	-0.580987361
AC092171.2	-0.575133009
AC080038.3	-0.572188729
SNRK	-0.564671325
NRG4	-0.558367163
KIAA1614	-0.540461178
NAV2-AS1	-0.516495273
IPPK	-0.514546847
SREK1	0.501683382
FBXL3	0.503361121
ERAP1	0.510214391
AL136311.1	0.511706198
PXK	0.516799307
STAG2	0.519255224
AP001496.3	0.531647196
PFKFB2	0.548207937
AL590627.1	0.54867793
ZNF135	0.564103835
RNF169	0.581682158
CASK-AS1	0.582380731
NEK5	0.586596231
AL731566.1	0.592894966
GANC	0.602748077
RNF19A	0.627026603
PIGF	0.630248185
DLG5	0.637956919
DDR1	0.64411491
SMG7-AS1	0.646381923
PLEKHG1	0.647145155
AC036103.1	0.650499517
DPH1	0.663221794
AL353593.2	0.663264816
AL049829.2	0.665821701
ATM	0.668470905
ODF2-AS1	0.671236377
GNPTAB	0.675979907
VPS13C	0.6774822
HIST1H4I	0.68228335
AC005520.5	0.683596209
TTBK2	0.684220162
AC091153.4	0.685573704
SLC44A1	0.691357153
CMTM7	0.694320835
AC007938.2	0.700870074
CEP290	0.70472518
FAM13A-AS1	0.704933291
MICAL1	0.71796338

SLC49A3	0.71908053
CEP72	0.720450769
COL6A2	0.72224681
ZC2HC1C	0.728126236
ARAP1-AS1	0.728554344
AL035448.1	0.734207973
CELSR2	0.734269909
ISOC1	0.738242903
BRWD3	0.739869102
NSMAF	0.740197763
TFF3	0.7548034
SLAMF8	0.754955674
CKB	0.755438637
RAG2	0.761984607
INE2	0.771095858
YAP1	0.786970747
GDPD2	0.78765185
GRIA1	0.790676612
BMP1	0.791080229
AL391421.1	0.794593807
CDH10	0.796621587
KIF1B	0.798882984
PLEKHA5	0.80365623
CFAP69	0.805735629
ANKRD61	0.807514958
SIK2	0.810033536
AC021504.1	0.814152204
TMEM87A	0.816092978
FAM227B	0.818208705
AP000873.3	0.818308739
TBC1D2B	0.822299206
AC079329.1	0.822878281
EPHX1	0.826755632
DNAH11	0.831503131
SMPD3	0.831543476
COG6	0.835102494
AL391839.2	0.837241423
HLTF-AS1	0.839801934
ZNF573	0.849286819
ZKSCAN1	0.849448288
EMILIN3	0.860434213
RIOX2	0.86077812
TLDC2	0.870487268
GPRC5B	0.873514762
PROX1	0.873691236
NBPF1	0.875310339
KIAA1328	0.879122055
AC078795.2	0.888041584

FLNB-AS1	0.889735317
POT1	0.900494873
AL158835.2	0.906574079
CDKN1C	0.907624882
FAM166A	0.9231997
PDE3A	0.936706026
C1GALT1	0.937201637
PPM1E	0.939074041
LAMA5-AS1	0.946100649
CRYBG3	0.946601365
THSD4	0.951040859
NBPF10	0.951824278
ARFGEF1	0.952348375
AP000942.2	0.959337162
PCDH7	0.963125237
KIAA1324L	0.968541577
C14orf93	0.971438153
SEMA6A	0.981731092
CD36	0.984950773
AC015563.2	0.990539842
CENPW	0.990928964
PCDHA10	0.998024939
RBM24	0.999069592
ZNF703	1.00141371
GIPR	1.002251248
USP25	1.002516851
LDB2	1.003818557
AOX1	1.008079357
SLC8A1-AS1	1.016369182
AC003986.1	1.019771565
CNTLN	1.02309808
RGS9	1.024101387
F13A1	1.024238048
PRSS54	1.026681885
CAMK1D	1.026740195
AL139398.1	1.029705109
GHDC	1.03428059
UFSP2	1.040595376
CDO1	1.054440753
SERPINF2	1.056029518
CERNA1	1.062032646
RPS26	1.070512581
ZNF518A	1.078988152
AP000688.1	1.079654721
BOC	1.079665686
AC016597.1	1.082302474
LMAN1	1.094270342
CBLN1	1.102847227

CCDC13-AS1	1.105910734
ENPP2	1.109114827
AGPS	1.113765639
LRRK2	1.117634522
SESTD1	1.124239781
AL033397.2	1.12991896
SGSM1	1.133138102
AC069444.1	1.141724322
CFTR	1.145089003
AC078795.3	1.145406973
AL078601.2	1.145992418
PITPNM3	1.155592447
AC099795.1	1.156753566
NEK7	1.169628458
ENPP1	1.171113552
MAP7	1.17405496
AC098851.1	1.181519658
AC012485.1	1.181946771
AC104964.4	1.182197684
TPT1-AS1	1.191267991
TAGLN2	1.196683888
PRRG3	1.201319166
ARHGEF26	1.201685539
AC073869.1	1.205167094
CLCN5	1.205872017
IMPA2	1.206951495
SYNE1-AS1	1.209514327
UBXN10	1.212162358
HCG11	1.216720169
GPR37	1.223946314
AC078883.1	1.22761555
PISD	1.228235695
CCDC114	1.230285914
CDH23	1.230609653
LYST	1.233354945
AC079385.2	1.236565767
AL133367.1	1.239252143
RAB38	1.241961403
AC126755.1	1.243512859
SERPINI1	1.245927232
ATPAF2	1.246643962
IL1RAP	1.251000634
DMD	1.254644293
AC067930.5	1.256939402
MYO3A	1.272284791
PIAS1	1.280701164
DLC1	1.282209653
NF1	1.285944703

FAM196A	1.294109232
PPP1R1B	1.297429402
AL050331.2	1.307909251
SRPX2	1.310721104
NEK9	1.311458998
AL137804.1	1.313663757
SNRPCP3	1.31391476
FHIT	1.327661589
TUSC1	1.333463067
STARD5	1.336522925
SLC29A1	1.339732287
AC007255.1	1.340435974
GALK2	1.344535009
CEP126	1.349177878
SPATA17-AS1	1.349676489
AC010969.1	1.357255739
HMGN5	1.357257863
AC093535.1	1.35795144
KCNH5	1.35841936
GAS8-AS1	1.362144543
JPH1	1.362733322
MS4A8	1.373829359
KLF5	1.376901457
SLC37A1	1.380998939
LINC00602	1.390274936
ROR2	1.390649001
VILL	1.390846164
AC233296.1	1.391230104
CD200	1.412054634
MPPED2	1.416981704
HCG4P5	1.420913868
KCNH2	1.427642807
TAF1C	1.430864308
SPOCK2	1.438576888
KCNH7	1.440124928
PHEX	1.445302196
OTX2	1.448993838
RASA4B	1.454917778
AC104964.1	1.458453811
LRP1B	1.46266518
MYO1D	1.466027247
TMC5	1.467466516
AC097515.1	1.471739118
ADAMTS2	1.472682441
NR4A2	1.472916944
AL354733.2	1.474988082
ITGA11	1.486227703
CFAP44-AS1	1.486759925

HNMT	1.491436871
MMRN1	1.491732151
PPP1R3C	1.495001375
AC002398.2	1.497403645
TC2N	1.497788917
ADAMTS12	1.499765402
AC027097.2	1.503260122
SELENBP1	1.507321747
IQCA1	1.511702041
AC092134.1	1.513957005
AC002064.2	1.518778872
LINC00954	1.519534811
TBX18	1.526559965
AQP3	1.526900324
AC008011.2	1.539609663
CHN2	1.545029116
LURAP1L-AS1	1.547407329
AC092641.1	1.555924014
AC079385.3	1.56132382
GPR156	1.568226846
AL354733.1	1.573696039
AC078883.3	1.574769398
LGI3	1.57785541
ATP8B4	1.588996452
RSPO2	1.58950254
C3orf67	1.593567654
AC078883.2	1.596349699
MARCHF7	1.607444219
PTCHD1	1.618098879
CFC1	1.623884056
AC078882.1	1.634189337
KLHL41	1.642882832
TPPP3	1.674907251
LRMP	1.676757499
AC027097.1	1.678203913
AC130456.3	1.682145314
NPAS2	1.69039847
LPIN3	1.705654797
DISP1	1.715665786
LINC02381	1.735066527
ALK	1.739713786
IQCA1-AS1	1.742394106
CR392000.1	1.74977857
ARHGEF11	1.751183968
HTR2C	1.757666734
MMP11	1.760325686
TTC14	1.764491913
AP003032.2	1.779615827

KCTD12	1.787129069
RBFADN	1.796527465
FGL2	1.813646845
EDARADD	1.822491378
INHA	1.824553995
CDCA7L	1.824877737
CTNNA3	1.829503353
AP000320.1	1.842662689
COL16A1	1.849028175
AC092142.1	1.853481738
AC008114.1	1.863672584
FER1L4	1.87711173
LINC01123	1.880077703
AL590560.2	1.887538935
ZNF385B	1.898970048
SBSPON	1.914715543
CDH7	1.923237802
AC008073.2	1.925210985
NUDT7	1.929231415
ADTRP	1.936343765
ACCS	1.943975274
PABPC4L	1.944952776
HHATL-AS1	1.953087025
DEPTOR	1.955550048
SIX3	1.96131923
ZXDC	1.967047594
AP000560.1	1.974976266
MYO7A	1.997942984
DCAF12L1	2.005836151
SERPINA12	2.014251562
RPL21P43	2.015186346
SLC17A9	2.015909681
AL049697.3	2.02148983
ELN	2.035668623
AC010255.2	2.041999071
DGKG	2.069764721
SCGB1B2P	2.073074629
UNC13C	2.089156192
HIST1H2BC	2.102336041
LINC02052	2.106057637
AGTR1	2.109983123
MUC5B	2.119744076
BCAS1	2.119984266
AC005682.1	2.137416298
KAAG1	2.153158255
ABCB1	2.158917745
RIMS1	2.160244779
FYB2	2.162022742

LINC00467	2.17869866
IL1A	2.182675625
AC005209.1	2.187028314
PDZD3	2.196396857
AL512625.1	2.201508388
MUC15	2.220391179
HTR1E	2.239222995
ALPK3	2.246628959
AC016757.1	2.259080379
HEY2	2.260432402
C1orf158	2.26272048
PTCHD1-AS	2.264691523
LTBR	2.272686489
CALCRL	2.274508779
AL353572.1	2.28814172
GABRG1	2.319465926
AC097468.1	2.332943279
CYP11A1	2.384275767
AC025038.1	2.385697248
NNMT	2.392031337
AC130456.4	2.413236239
TTR	2.415955986
SMOC1	2.431529766
SLC38A4	2.432177856
SLAMF9	2.434916421
OTOF	2.438065045
COL8A2	2.440391242
CILP	2.4429999
PI16	2.453284328
TRIM58	2.466120317
NKX3-1	2.477162127
AL021391.1	2.497245462
AL355812.1	2.514887682
AP005120.1	2.517407272
GZMA	2.5435967
CNMD	2.569256265
EGFL6	2.601010737
SLC22A8	2.653853247
AC104809.1	2.682449831
PRKAG3	2.743168102
CCRL1P1	2.784860628
AC126323.6	2.795811377
AL392003.2	2.827602631
LINC02020	2.827769923
AC005871.2	2.853124899
LINC02389	2.854986809
SSTR5-AS1	2.899796755
CLDN5	2.901522745

AC079360.1	2.908736275
HSPA8P7	2.912099073
SLC28A3	2.92667504
EPS15P1	2.935332514
RAB37	2.966696327
SPDEF	2.985989169
LINC02056	3.012624473
LINC01361	3.074152935
RF00019	3.081729229
LINC01257	3.088816575
AC061979.1	3.096446458
AL356134.1	3.10434782
ACAN	3.105117014
NPFFR2	3.142334439
LINC00654	3.167403489
AC138819.1	3.200121296
AL512625.3	3.202513781
AC098476.1	3.217874585
FER1L6	3.33832231
TTLL10-AS1	3.348024901
AC005281.1	3.443988178
PZP	3.460660282
CRACR2A	3.465026778
AC005831.1	3.671035995
CCDC68	3.723613334
AL161618.1	3.749869823
LINC01537	3.891135409
DGAT2L6	3.9650696
LINC02208	3.975688856
MUC5AC	4.086899719
AC091062.1	4.458167326
AP001999.1	4.715998118
CYP26C1	4.852844974
AC097493.2	4.895581365
PRPH2	5.390830869
SMIM31	5.725410845
MYO5BP2	6.307212632

Supplementary Table 13. DEGs between DA neurons derived from PD patients with E326K-*GBA1* mutations compared to control neurons and DEGs between DA neurons derived from sPD patients compared to control neurons.

Comparison	Original	Bonferroni	Holm_Bonferroni	Benjamini_Hochbe
Control-sPD Na	0.003	0.054	0.039	0.0041538
Control-GBA1 Na	0.0028	0.0504	0.0392	0.0036
Control-GBA1 Fast potassium	0.5201	1	1	1
Control-sPD Fast potassium	0.5597	1	1	0.93184
Control-GBA1 Slow potassium	0.4515	1	1	1
Control-sPD Slow potassium	0.3523	1	1	0.90591
Control vs GBA1 PD (EPSC Rate)	0.0389	0.7002	0.389	0.07002
Control vs sPD (EPSC Rate)	0.11	1	0.99	0.22
Control vs GBA1 PD (EPSC Amplitude)	2.244E-05	0.000404	0.00040397	2.24E-05
Control vs Sporadic PD (EPSC Amplitude)	0.0008995	0.016191	0.013492	0.0010794
Control vs GBA1 PD (Total spikes)	4.986E-05	0.0008975	0.00084764	5.28E-05
Control vs Sporadic PD (Total spikes)	0.008	0.144	0.088	0.013091
Control vs GBA1 input conductance	0.8613	1	0.8613	1
Control vs sPD input conductance	0.6791	1	1	1
Control vs GBA1 capacitance	0.2666	1	1	0.59985
Control vs sPD capacitance	0.842	1	1	1
Control vs GBA1 large epsc rate	0.000061	0.001098	0.000976	0.000069
Control vs sPD large epsc rate	0.0042	0.0756	0.0504	0.0063

Supplementary Table 14. Multiple hypothesis corrections for the electrophysiological data.
